# Non-Dikarya Fungal Clades Are Everywhere: What 18S rRNA Gene Metabarcoding Reveals About Cross-System Distribution of Fungi

**DOI:** 10.1007/s00248-025-02642-w

**Published:** 2025-11-24

**Authors:** Sofiya Bondarenko, Aleix Obiol, Emilio O. Casamayor, Ramon Massana

**Affiliations:** 1 Department of Marine Biology and Oceanography, Institut de Ciències del Mar (ICM-CSIC), Passeig Marítim de La Barceloneta, 37-49, 08003 Barcelona, Catalonia Spain; 2 Ecology of the Global Microbiome-Department of Ecology and Complexity, Centre d’Estudis Avançats de Blanes (CEAB-CSIC), Catalonia, Spain

**Keywords:** Amplicon sequence variants (ASVs), Chytridiomycota, Fungal taxa, Non-Dikarya fungi, Rozellida, Aphelidea

## Abstract

Non-Dikarya fungi remain poorly characterized due to their cryptic morphology, cultivation difficulties, and limited representation in reference databases. To investigate their diversity and environmental distribution at a global scale, we reanalyzed over 6000 environmental samples using metabarcoding targeting the V4 region of the 18S rRNA gene, encompassing marine, freshwater, soil, hypersaline, polar, and other habitats. We constructed reference phylogenetic trees based on near full-length 18S rRNA gene sequences to enable accurate placement of short-read amplicon sequence variants (ASVs). This approach yielded robust classification at the phylum level and provided finer-scale clade resolution within major non-Dikarya groups. We delineated precise clades within Chytridiomycota, Microsporidia, Rozellida, and Aphelidea, and unveiled several novel ones. Our results show strong ecological structuring of fungal communities across habitats, with inland systems harboring greater fungal abundance and broader phylogenetic diversity than marine systems. Non-Dikarya fungi were consistently detected across diverse environments, including extreme habitats such as hypersaline lakes, deep sediments, and polar regions, where they were often the dominant fungal taxa. Although most ASVs tended to occur in a limited number of ecologically related habitats, phylogenetically related ASVs within the same clade were often adapted to different environments, indicating ecological diversity within clades. Our findings underscore both the ecological relevance and the cryptic diversity of non-Dikarya fungi in globally distributed environments, including extreme ones. Improved taxonomic resolution and broader reference dataset coverage are required to fully integrate these newly characterized lineages into fungal systematics and environmental surveys.

**Supplementary Information:**

The online version contains supplementary material available at 10.1007/s00248-025-02642-w.

## Introduction

Fungi are among the most crucial biological components of ecosystems worldwide. Numerous studies have shown their prominent role in nutrient cycling, particularly through their ability to degrade even the most complex organic compounds [1, 2]. In addition, fungi influence ecosystem dynamics by forming symbiotic relationships, many of them parasitic on a wide array of hosts, and contribute to the stability of microbial communities thanks to their adaptability to a wide range of environmental conditions [3–5]. Despite their ecological importance, many aspects of fungal biology remain poorly understood. These “dark matter” fungi [6] are notoriously difficult to cultivate and exhibit limited morphological differentiation. Their sparse representation in reference datasets greatly hinders molecular identification and constrains taxonomic resolution, especially when environmental sequences are allocated within poorly characterized lineages [7]. This is especially true for non-Dikarya fungi [8] such as Chytridiomycota, Microsporidia, Rozellida, and Aphelidea.

Chytridiomycota include primarily unicellular fungi, though some species form simple multinucleate structures such as rhizomycelia [9]. Their life cycle typically includes a walled sporangium in which numerous posteriorly uniflagellate zoospores develop [10]. These fungi exhibit broad ecological versatility, and their ecological roles range from saprotrophy to parasitism on algae and other protists or on animals [9, 11]. Chytridiomycota contribute significantly to aquatic food webs through the mycoloop, where their zoospores link phytoplankton to zooplankton, facilitating carbon and nutrient transfer [12]. They also play a role in the mycoflux, influencing carbon cycling during phytoplankton blooms [13], thus highlighting their ecological importance in energy flow and microbial interactions.

According to recent studies, Microsporidia and Rozellida may represent two distinct sister groups [14], although other studies—and the classification generally followed by the mycological community—support a continuum between them and unite them within Rozellomycota [15, 16]. In this study, we decided to consider Microsporidia as a sister group to Rozellida, incorporating many lineages previously classified under the phylum Rozellomycota (= Cryptomycota) [14]. These are putatively considered true microsporidians based on the presence of key morphological traits—thick-walled spores equipped with a polar filament apparatus and a merogonial stage during intracellular development [14], although this is still based on too few characterized species. It should be noted that in the present study, when we refer to Microsporidia, we only consider short-branch microsporidians (SB-Microsporidia). As compared with the classical long-branch microsporidians, which typically escape metabarcoding detection, SB-Microsporidia retain more conserved rRNA sequences, are detectable by standard metabarcoding, and exhibit genomic features characteristic of fungi. Recent studies indicate that SB-Microsporidia are broadly distributed across diverse environments, with a notable preference for freshwater and terrestrial habitats [17]. Microsporidia are best known as intracellular parasites of invertebrates and vertebrates, but they also occur as endosymbionts or even hyperparasites of ciliates and other protists [14].

Rozellida are endoparasitic fungi that grow as naked protoplasts within host cells, lacking a cell wall during intracellular stages [18]. Their flagellated zoospores are motile but do not contain a polar filament, unlike those of many Microsporidia [14]. Their widespread environmental distribution is likely linked to parasitism—or hyperparasitism—of diverse heterotrophic hosts such as chytrids, oomycetes, and potentially other understudied protists [11, 19, 20].

Aphelidea are known as endoparasites [21] and retain features not typically associated with true fungi, such as phagocytosis. Nonetheless, they are currently considered among the earliest-diverging fungal lineages, although their exact phylogenetic placement remains unresolved and may be subject to future revision [22]. Aphelids have a distinctive life cycle involving flagellated, amoeboid, or dual-motility zoospore stages, depending on the genus. They have been recorded primarily in freshwater environments, but some genera are associated with coastal marine habitats [10, 23]. Aphelids are known as parasites of autotrophs, such as green algae and diatoms.

Although several ecological, morphological, and physiological traits have been described for non-dikaryotic lineages, these insights are based on a limited number of cultured and morphologically characterized representatives. While often these representatives are phylogenetically scattered across broader clades, it still remains unclear whether the same ecological strategies and morphological features apply to the numerous taxa detected exclusively through sequencing approaches. This uncertainty is particularly exacerbated for Microsporidia, Rozellida, and Aphelidea, fungal groups in which the diversity may be underestimated due to sparse taxon sampling.

Recent advances in high-throughput sequencing technologies have improved our ability to detect fungi across diverse ecosystems—from terrestrial to marine, and even extreme environments such as polar lakes [24–26]. While long-read sequencing [20], metagenomics [27], and single-cell sequencing approaches [11] offer promising insights, the initially established method of short-read metabarcoding still serves as a major source of fungal diversity data for massive environmental surveys. While the ITS region is commonly used in fungal metabarcoding because of its finer taxonomical resolution, the 18S rRNA gene—particularly its V4 region—enables broader ecological comparisons by capturing a wide range of eukaryotic taxa within the same community. Moreover, it is particularly suited for studying non-dikaryotic fungal taxa [28]. Several 18S rDNA metabarcoding databases are now publicly available, including EukBank [29] and metaPR2 [30]. Together with an additional curated dataset maintained by our own research group, these resources comprise thousands of samples derived from a wide range of environments.

In this study, we investigated the diversity and environmental distribution of fungi across a broad range of systems, including inland, marine, and extreme habitats such as polar and hypersaline lakes. By reclassifying 18S rRNA amplicon gene sequences from public and curated datasets with an updated phylogenetic framework, we aimed to improve taxonomic resolution and unveil patterns across the fungal tree of life. Special emphasis is placed on non-Dikarya lineages, which are frequently misclassified or overlooked in conventional metabarcoding studies, yet represent a significant and relevant fraction of fungal biodiversity and function.

## Methods

### Trees for Taxonomic Assignment

Fungal sequences for the reference Opisthokonta tree were obtained from previously published studies [18, 31]. Additionally, sequences belonging to Opisthokonta were extracted from in-house datasets of 18S V4 environmental ASVs (see below) and queried against the PR2 database [32] using BLAST [33] to identify long reference sequences closely related to environmental ASVs. We also selected 12 outgroup sequences from the PR2 database. Sequence ends were trimmed if needed according to the typical 18S rRNA gene length. Short reference sequences (< 700 bp) were removed. Sequences were clustered using VSEARCH 2.28.1 at 99% identity for fungi and at 95% for metazoans. Alignment was carried out with MAFFT version 7 using the FFT-NS-i algorithm. Misaligned metazoan sequences were manually removed and the alignment was redone. A maximum likelihood tree was constructed using IQ-TREE 2.0.6 with the SYM + R10 model.

For more precise taxonomic resolution, three separate reference trees were constructed for the most abundant non-Dikarya lineages: (i) Chytridiomycota, (ii) Microsporidia plus Rozellida, and (iii) Aphelidea. Long reference sequences (≥ 700 bp) were selected from Bass et al. (2018) [14] and Seto et al. (2023) [11], and additional sequences were retrieved by performing a BLAST of the classified ASVs against the NCBI database (retrieval date 28.02.2025). Alignment was performed with the automatic algorithm in MAFFT, and phylogenetic trees were performed with IQ-TREE using different substitution models, as specified in the Figure legends. Support values for all trees were estimated using 1000 SH-aLRT and 1000 ultrafast bootstrap (UFBoot) replicates.

### Building V4 18S rRNA Gene Metabarcoding Database

For our investigation, we merged three metabarcoding datasets targeting the V4 region of the 18S rRNA gene, obtained with the universal eukaryotic primers described by Stoeck et al. [34] or with a single modification [35]. First, the publicly available EukBank dataset [29], comprising 12,672 samples collected from a wide variety of terrestrial and marine habitats. Second, the metaPR2 database [30] from which we extracted 287 coastal marine samples from the Ocean Sampling Day project. Third, metabarcoding data of 495 marine and inland samples prepared by the Ecology of Marine Microbes (EMM) research group (ICM-CSIC). Illumina reads are available in ENA for projects with coastal (PRJEB63614, PRJEB64697, PRJEB97527) and offshore (PRJEB44683) samples from the Mediterranean Sea, oceanic bathypelagic samples (PRJEB45014), samples from the Antarctica Peninsula (PRJEB97758), inland samples from the Delta del Llobregat, Catalonia, Spain (PRJEB97757), and saline sediments and waters from the Monegros Desert, Zaragoza, Spain (PRJEB97742).

To construct the merged fungal dataset, we removed samples that had any of these limitations: (i) had been sequenced with Roche 454 technology, (ii) contained fewer than 10,000 reads, (iii) had missing or incorrect geographic coordinates, (iv) derived from host microbiomes, and (v) were part of time-series projects. We classified all samples as either polar or non-polar, considering polar those located above 66.33°N or below 66.33°S. Inland water samples were classified based on salinity and/or the EukBank classification into three categories: freshwater (0–0.9% salinity), brackish/saline water (1.0–4.9%), and hypersaline water (5.0–40.0%). For marine samples, we further categorized them as surface or deep depending on the sampling depth, with 200 m as the threshold. To distinguish between coastal and oceanic marine samples, we used two criteria: proximity to the shoreline and bottom depth. The shortest distance from each sample to the nearest coastline was calculated using simplified Natural Earth coastline data and the sf package v1.0–19 [36] in R version 4.3.2 [37], applying the Mollweide projection. Bottom depth was derived from NOAA bathymetric data using the marmap package [38]. Samples were classified as coastal when they met the following two conditions: located within 5 km of the coastline and bottom depths < 200 m. Marine samples that met only one of the two criteria (e.g., sites close to shore but with a bottom deeper than 200 m) were excluded from further analysis. The final dataset included 6,154 samples (5457 from EukBank, 209 from metaPR2, and 488 from the EMM group). Most of the excluded samples were removed during the classification of coastal versus oceanic environments, as well as during the removal of microbiome and time-series samples, and those with low read counts.

From the taxonomy table, already classified to group-level taxa, we removed ASVs shorter than 300 bp as well as those assigned to Metazoa or Streptophyta, to obtain the subset of microeukaryotes. We then recalculated the total number of reads per sample. Sequences assigned to Opisthokonta were reclassified by phylogeny-aware alignment with PaPaRa 2.5 [39] and taxonomic placement with EPA-ng 0.3.8 [40] and gappa 0.8.4 [41]. For ASVs that lacked clear taxonomic placement, we constructed additional phylogenetic trees with IQ-TREE version 2.0.6 [42], by adding these ASVs to the reference alignment using MAFFT [43]. ASVs that still could not be clearly assigned were labeled as InSedOpisthokonta or InSedFungi if they clearly fell within the fungal clade. Finally, we removed ASVs that occurred in only one sample, resulting in a final dataset with 9835 fungal ASVs.

### Data Analyses

All data analyses were performed in R version 4.3.2 [37], using the tidyverse package version 2.0.0 [44], and the phyloseq package version 1.46.0 [45] as implemented in speedyseq [46]. Ordination analyses were carried out using the vegan package version 2.6.10 [47] on the table of ASVs counts for all fungi. We first computed the average Bray–Curtis dissimilarity matrix from 100 iterations using the avgdist() function with a subsampling of 100 reads. This matrix was then used to perform non-metric multidimensional scaling (NMDS) with the metaMDS() function. Heatmap analyses were conducted using the ComplexHeatmap package version 2.18.0 [48] based on matrices of log₁₀-transformed relative read abundances, with the smallest positive value added as a pseudocount. To make the heatmaps easier to read, for Chytridiomycota and Microsporidia plus Rozellida, we selected only the most abundant and frequent ASVs. For each ecosystem, we calculated how often each ASV appeared (the percentage of samples where it was present), then averaged these values across all ecosystems. We also calculated the relative abundance of each ASV, but only among the samples where it was detected. For the final analyses for Chytridiomycota, we took 388 ASVs with occurrence > 0.7% and relative abundance > 0.1%; for Microsporidia plus Rozellida, we took 363 ASVs with occurrence > 1% and relative abundance > 0.1%; and for Aphelidea, we took all 227 ASVs regardless of their abundance and occurrence.

## Results

### Backbone Reference Tree of Opisthokonta

**Fig. 1:**
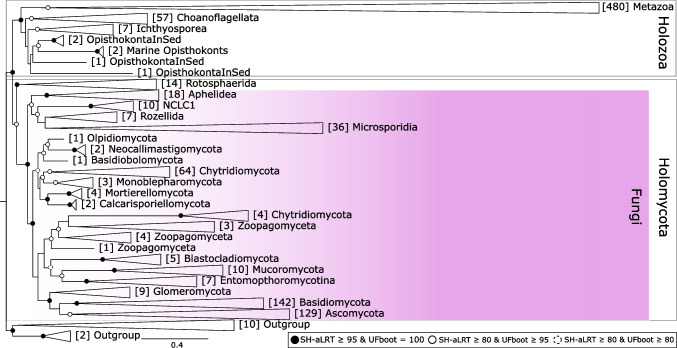
Reference tree of fungi and other Opisthokonta based on long 18S rRNA gene sequences collapsed by groups (see Fig. S1 for the uncollapsed tree). The tree was built using IQ-TREE with the SYM + R10 model using 1031 Opisthokonta sequences (clustered at 99% identity, except 95% for Metazoa) and 12 outgroup sequences. Support values were estimated using 1000 SH-aLRT and 1000 UFBoot replicates. The number of sequences within each group is denoted in brackets before the group name

For proper taxonomic assignment, we constructed a reference backbone tree of Opisthokonta based on long 18S rRNA gene sequences (Fig. 1). The resulting topology was consistent with the standard phylogeny of this supergroup and clearly resolved the separation between Holozoa and Holomycota, as well as among fungal lineages and Rotosphaerida. We were able to split all fungi into 18 groups, including Ascomycota and Basidiomycota that form altogether Dikarya, and 16 non-Dikarya fungal groups. All fungal groups showed strong support values, with the exception of Zoopagomyceta and Chytridiomycota, which appeared as non-monophyletic groups in the tree, and Rozellida and Microsporidia, which showed lower internal support due to a few divergent sequences lacking environmental analogs in subsequent phylogenetic reconstructions.

### Fungal Habitats in the Global Metabarcoding Dataset

**Fig. 2:**
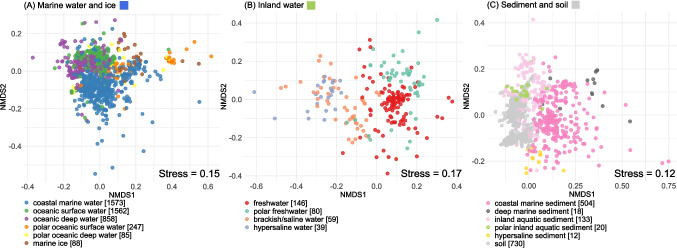
NMDS of samples based on fungal ASVs using Bray–Curtis dissimilarities. Separate NMDS plots were prepared for marine water and ice samples (**A**), inland water samples (**B**), and sediment and soil samples (**C**). Numbers in brackets indicate the original number of samples in the corresponding habitat. For the NMDS analysis, we considered only those samples that contained a minimum of 100 fungal ASV reads

We categorized all samples from our global dataset into 16 habitat types. The geographic origin of samples was illustrated on separate world maps for each habitat (Fig. S2). To investigate how these habitats differ in fungal community composition, we performed non-metric multidimensional scaling (NMDS) based on fungal ASV profiles. We performed three separate plots for samples derived from marine water and ice (Fig. 2A), inland water (Fig. 2B), and sediment and soil (Fig. 2C). The separation of samples among these three categories was supported by the NMDS plot with all samples (see Fig. S3), which showed a particularly clear differentiation between marine water and ice versus sediment and soil. Conversely, inland water samples showed partial overlap with marine water, likely reflecting the influence of estuarine systems.

The ordination plots showed a clear separation between coastal marine water and other marine water types (Fig. 2A). Within inland water, freshwater and polar freshwater showed more or less distinct profiles, whereas brackish/saline water and hypersaline water clustered together (Fig. 2B). Within the third category, coastal marine sediments formed a distinct cluster, separate from deep marine sediments (Fig. 2C). Soil, inland aquatic sediments, hypersaline sediments, and polar inland aquatic sediments also formed more or less separate groups. Overall, most of the 16 defined habitats displayed characteristic and well-differentiated fungal communities.

### Contribution of Fungal Groups Across Habitats

**Fig. 3:**
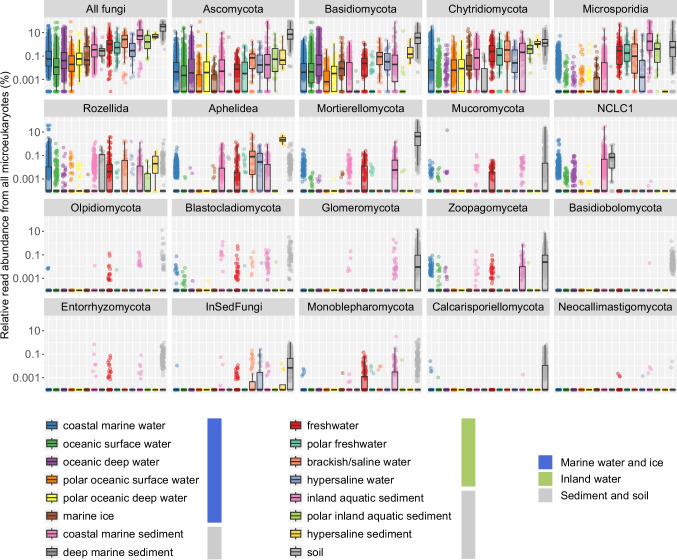
Distribution of all fungi and fungal groups across habitats. Each dot represents the pooled relative read abundance of all ASVs assigned to a given group within a sample. Values are shown on a logarithmic scale, with the lowest positive value in the dataset used as a pseudocount. Boxes in plots represent the interquartile range (IQR), with the median line inside. Whiskers extend 1.5 times the IQR, and individual points beyond this range represent outliers

The relative abundance of fungi from all targeted microeukaryotes (Fig. 3, first panel) varied substantially across environments ranging from undetectable levels in some marine samples to > 98% in a soil sample. On average, fungal abundance across all habitats was approximately 5%, with inland environments—such as freshwater and soil—consistently showing a higher proportion of fungi than marine habitats.

**Fig. 4:**
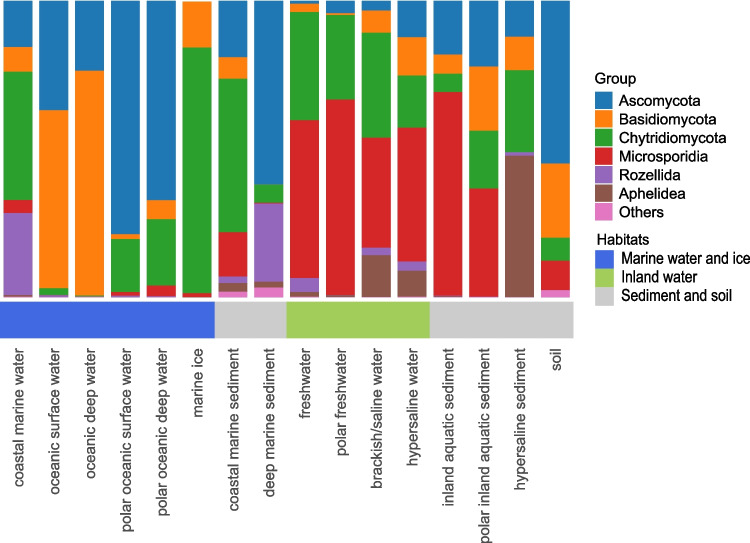
Relative read abundance of the main fungal groups in various habitats. Relative read abundance was calculated from all sequences assigned to fungi, with the most relevant groups shown individually and the remaining groups merged into the “Others” category

The relative composition of major fungal groups showed that coastal marine waters differed notably from oceanic surface and deep waters (Fig. 4, see Fig. S4 for more details). While the latter were dominated primarily by Ascomycota and Basidiomycota, coastal waters also showed a substantial presence of Chytridiomycota and Rozellida. Similarly to coastal marine waters, polar marine environments exhibited a large contribution of Chytridiomycota, mostly accompanied by Ascomycota and with a minor contribution of Basidiomycota. Inland habitats had distinct fungal community compositions, characterized by high proportions of Chytridiomycota and Microsporidia, alongside the minor presence of Ascomycota and Basidiomycota. Aphelidea were a notable component of brackish, saline, and hypersaline habitats. Overall, each habitat showed a distinct contribution of fungal lineages, and in many of them, non-Dikarya lineages dominated.

A more detailed analysis of the distribution of all fungal groups across habitats confirmed, on the one hand, the widespread presence of Ascomycota and Basidiomycota, and on the other hand, the ubiquitous environmental distribution of several non-dikaryotic fungal lineages (Fig. 3). Specifically, Chytridiomycota was broadly distributed but nearly absent from oceanic surface and deep waters. Microsporidia, Rozellida, and Aphelidea appeared more associated with inland habitats, though they were also present in coastal marine sediments, and Rozellida notably occurred in deep marine sediments. Several other groups were less abundant and showed patchy distributions, primarily in inland environments. For example, Mucoromycota and Mortierellomycota were mostly found in soil or sediments, while Glomeromycota were almost exclusively detected in soil. Notably, the novel chytrid-like-clade-1 (NCLC1) group showed high abundances in various marine sediment types.

### Defining Clades in Non-Dikarya Fungal Lineages

For a deeper investigation of non-Dikarya fungal lineages, we focused on the four most prevalent groups in the global dataset: Chytridiomycota, Microsporidia, Rozellida, and Aphelidea. We first reconstructed phylogenetic trees using long sequences (> 700 bp) to capture the diversity within each group and to compare our findings with previously published studies. The resulting clades were evaluated against existing taxonomic frameworks, and novel clades were defined when necessary (Fig. S5, S7, S9). We then incorporated the most abundant and frequently detected ASVs into the reference alignments (or all ASVs in the case of Aphelidea), generating new trees that included both long and short sequences to enable classification of ASVs into the established clades. In several cases, the placement of ASVs revealed additional, previously unrecognized clades.

**Fig. 5:**
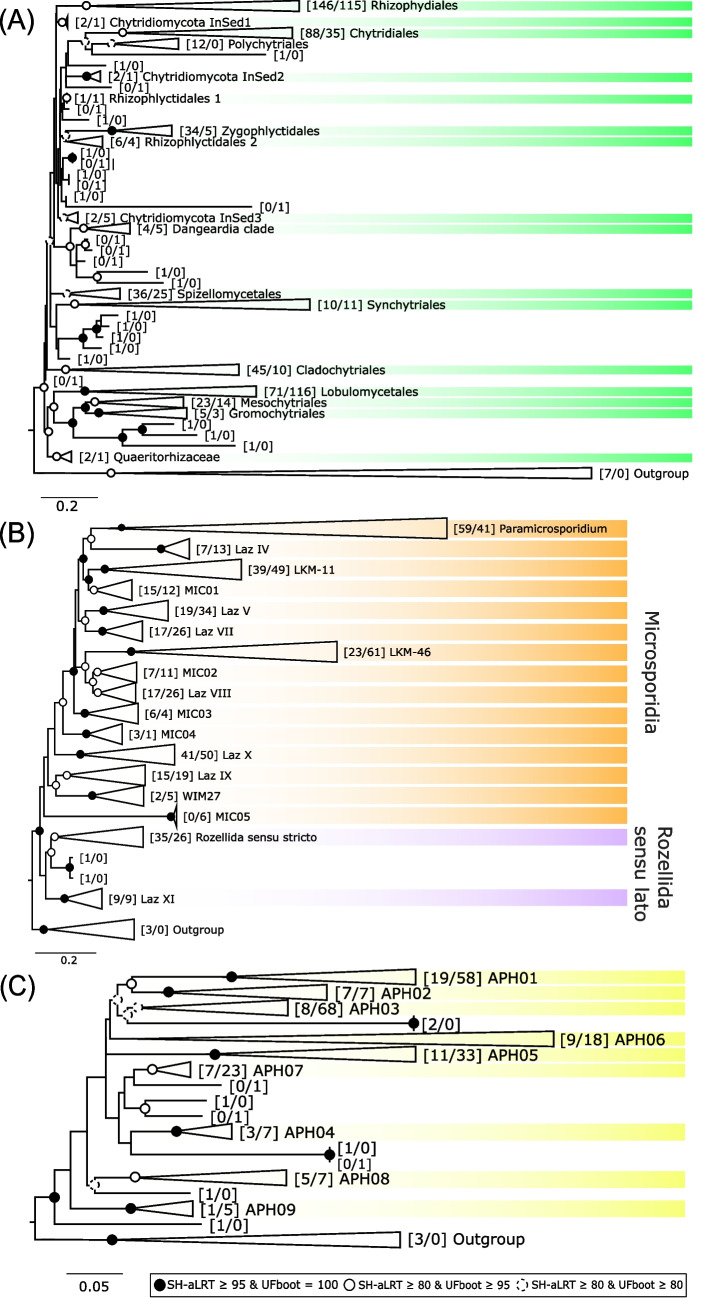
Collapsed phylogenetic trees with complete 18S rRNA gene reference sequences and ASVs for the fungal groups Chytridiomycota (**A**), Microsporidia plus Rozellida (**B**), and Aphelidea (**C**). In each tree, an alignment of complete reference sequences was first prepared and used to incorporate partial ASVs. For Chytridiomycota, ASVs present in more than 0.7% of samples and with a relative abundance greater than 0.1% were included; for Microsporidia plus Rozellida, the thresholds were 1% and 0.1%, respectively; for Aphelidea, all ASVs were included. Phylogenetic trees of the resulting alignment were constructed with IQ-TREE using different models: TIM2 + F + R10 in **A**, GTR + F + R7 in **B**, and SYM + R6 in **C**. The original uncollapsed trees can be seen in Figs. S6, S8, and S10, respectively. Support values were estimated using 1000 SH-aLRT and 1000 UFBoot replicates. Values in brackets represent the number of sequences in each clade (reference sequences/ASVs)

In Chytridiomycota, we delineated 18 clades (Fig. 5A), most of which corresponded to previously described orders such as Spizellomycetales and Rhizophydiales. Additionally, we identified three novel clades (Chytridiomycota InSed1–3), each supported by at least two near-complete environmental 18S rRNA gene sequences. In Microsporidia plus Rozellida (Fig. 5B), we also defined 18 clades. Thirteen of these corresponded to clades previously reported in the literature, four were novel and supported by at least two long reference 18S rRNA gene sequences (MIC01-04), and one clade (MIC05) was entirely new, lacking any long reference sequence. For Aphelidea (Fig. 5C), we analyzed all sequences and identified nine novel clades. Eight of these were supported by multiple long reference sequences, while one clade (APH09) was represented by a single long reference sequence.

### Habitat-Specific Distribution of Non-Dikarya Fungal Clades

**Fig. 6:**
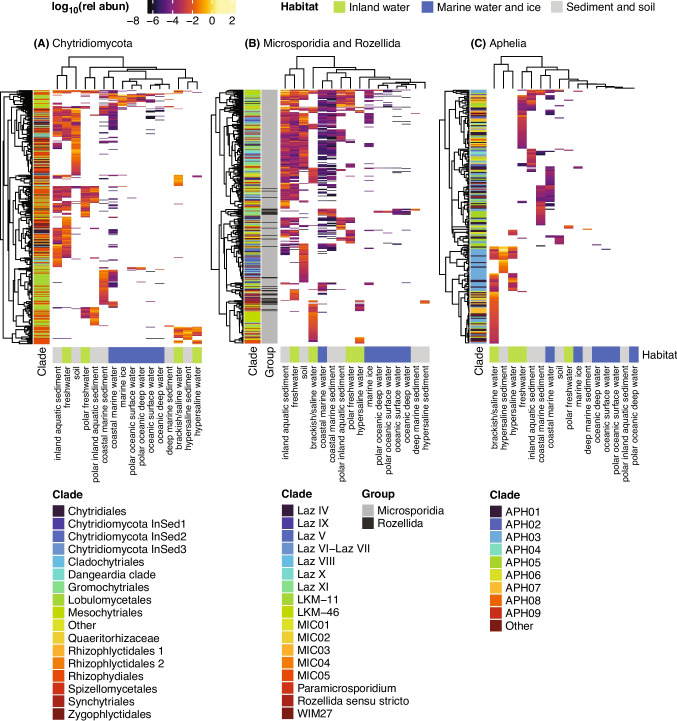
Heatmap of the distribution of dominant ASVs across habitats within Chytridiomycota (**A**), Microsporidia plus Rozellida (**B**), and Aphelidea (**C**). The ASVs considered dominant were the same selected to build the trees in Fig. 5. Both the ASVs and the habitats were clustered with Ward D2 method using Euclidean distances. Each ASV was colored by the clade it belongs

We selected the same set of dominant ASVs employed in the previous phylogenetic trees of the most widespread non-Dikarya groups of fungi and evaluated their distribution across the defined habitats with heatmaps. In these heatmaps, ASVs were grouped according to their habitat preference profiles, and habitats were also clustered based on their ASV composition. ASVs were color-coded by their phylogenetic clade assignment, enabling simultaneous comparison of taxonomic affiliation and ecological distribution (Fig. 6).

This analysis showed clear ecological structuring among habitat types. Broadly, water bodies—both inland and marine—tended to cluster with their sediment counterparts. Polar marine systems grouped together, as did polar inland environments. Soils tended to associate with freshwater and inland aquatic sediment habitats. Similarly, brackish/saline water, hypersaline water, and hypersaline sediments also formed distinct clusters, although some variability between groups remained. Coastal marine systems consistently formed a distinct cluster, separate from open oceans. Within this environmental framework, we found that individual ASVs, regardless of their clade affiliation, typically exhibited distinct habitat preferences—mostly being restricted to one environment or a few related environments. The overall distribution of ASVs within each clade was generally heterogeneous, with representatives of the same clade often occurring across a broad range of ecosystems.

An exception to this general trend was found in Aphelidea, particularly in clade APH03, whose ASVs consistently showed similar and narrow distribution profiles. Members of this clade were restricted to one, two, or all three of the following habitats: brackish/saline water, hypersaline water, and hypersaline sediment, and were almost absent from other environments. Interestingly, these three habitats formed a coherent cluster not only in Aphelidea, but also in Chytridiomycota, where several ASVs were exclusively associated with them. A similar but more nuanced pattern was observed in Microsporidia/Rozellida. For these two groups, hypersaline sediment was more distinct, while brackish/saline water and hypersaline water remained closely related in terms of ASV presence. Remarkably, the novel clade MIC05, which lacks long reference 18S sequences, was found exclusively in these two environments.

## Discussion

### 18S rRNA Gene Metabarcoding: Potential and Limitations

Our results demonstrate that near full-length sequences of the 18S ribosomal RNA gene (SSU rRNA) provide a robust phylogenetic framework that can be effective for resolving higher-level taxonomic relationships, such as those at the phylum level (Fig. 1), as well as for achieving finer resolution within non-Dikarya fungal lineages. These reference trees with near-complete sequences can then be used as a phylogenetic framework for the placement and classification of shorter environmental sequences targeting the hypervariable V4 region. Moreover, the phylogenetic structure is maintained when complete and short sequences are combined. For example, our phylogenetic reconstructions of the groups Aphelidea, Rozellida, and Microsporidia retain well-supported clade structures when short V4 sequences are added (Figs. 5B and 5C). Conversely, within more recently diverged groups such as Chytridiomycota (Fig. 5A), clade support tends to decrease, and certain taxonomic orders may appear polyphyletic or split across distinct branches even in the reference tree (Rhizophydiales) or when adding short sequences (Rhizophlyctidales). Despite these limitations, our results suggest that V4-based classification remains reliable at least up to the order level in this group.

These findings are consistent with previously published data. Specifically, while the small subunit (SSU) rRNA gene may lack sufficient resolution for distinguishing closely related taxa within groups such as Ascomycota and Basidiomycota—where sequences can be highly conserved even at the order level [49]—it remains a valuable marker for studying non-Dikarya lineages. In these lineages, the SSU rRNA gene often provides sufficient phylogenetic signal and is supported by a larger number of available reference sequences compared to the internal transcribed spacer (ITS) region [28]. Our results are consistent with the idea that the V4 region, when combined with complete 18S references, can approximate long-read metabarcoding, although this likely depends on the breadth and quality of available reference sequences.

We therefore consider the V4 region of the 18S rRNA gene particularly well suited for metabarcoding applications aimed at capturing fungal diversity at higher taxonomic levels and elucidating the evolutionary relationships of non-dikaryotic fungal taxa. Moreover, targeting this region facilitates the integration of fungal datasets into broader eukaryotic biodiversity surveys [50]. Phylogeny-aware approaches—such as constructing reference trees from full-length 18S rRNA gene sequences and placing environmental reads within them—further enhance the accuracy and resolution of taxonomic assignments [51].

### Phylogenetic Uncertainties in Non-Dikarya Fungal Groups

Although the taxonomic boundaries between Microsporidia, Rozellida, and Aphelidea remain unresolved, and discussions are currently ongoing on whether or not Microsporidia plus Rozellida form a single phylum, or whether aphelids belong within the fungal kingdom at all [15, 52, 53], our study does not attempt to revise current classifications. Instead, we mostly follow the existing frameworks outlined in recent literature [11, 14]. Thus, clade delineation within Chytridiomycota and Microsporidia largely derives from previous work (a few new clades were introduced here), while an entirely new within-group naming system was established for Aphelidea, as only one clade (GS16, corresponding to our APH03) had been previously described [54]. An exception to this is the use in this study of the broader term Rozellida, which also includes the clade NAMAKO-37 (= Laz XI; [14]), a group that phylogenetically falls within or is closely related to Rozellida. Similarly, it is important to note that we focused exclusively on short-branch Microsporidia (SB-Microsporidia), as long-branch microsporidians were excluded due to our V4 size-filtering threshold (> 300 bp) and primer biases. Nevertheless, a few sequences approximately 220 bp in length with similarity to long-branch Microsporidia were still recovered in our dataset.

Approximately 0.75% of ASVs in our dataset could only be assigned to the broad category “InSedFungi,” without further taxonomic distinctiveness. In addition, some fungal sequences may fall within unclassified Opisthokonta, reflecting the ongoing lack of phylogenetic resolution for some non-Dikarya lineages and the existing novel diversity remaining uncharacterized. These unresolved ASVs are not marginal as they occur across multiple environments and can be particularly abundant in soils (Fig. 3). The limited availability of well-anchored reference taxa—especially in groups such as Aphelidea—illustrates a broader bias. While significant progress has been made in fungal systematics, the classification of non-Dikarya lineages remains incomplete. Continued efforts toward standardized naming conventions and broader phylogenetic coverage will be essential to better integrate novel environmental diversity into fungal taxonomy and ecological interpretation. In this context, databases such as EUKARYOME [55], which currently include extensive long-read rRNA sequence data and cover a wide range of non-Dikarya fungi, represent an important step toward improving reference coverage and could substantially complement future taxonomic and ecological studies.

### Habitat-Specific Patterns of Fungal Diversity

According to metabarcoding data, fungi are present across a wide range of habitats, and their diversity is remarkable. In general, the relative abundance of fungi is higher in inland habitats than in marine habitats, highlighting the particularly important role fungi play in inland systems. Also, inland habitats host a broader range of non-dikaryotic fungal groups in addition to the nearly ubiquitous Ascomycota and widely distributed Basidiomycota—the most common are Chytridiomycota, Microsporidia, Rozellida, and Aphelidea, though not exclusively. The particular contribution of fungal groups varies substantially between habitats.

The environments with the lowest relative abundance of fungal sequences and the lowest fungal group diversity appeared to be oceanic surface and deep waters. In these habitats, the fungal community is dominated by Ascomycota and Basidiomycota. Conversely, coastal marine waters and sediments showed increased representation of Chytridiomycota, a group that is also abundant in polar marine ecosystems and marine ice, and that is well represented in inland habitats. Notably, even in soil environments, zoosporic fungi such as Chytridiomycota are common, confirming that even a transient water film could be enough for the dissemination of their zoospores [10]. These findings align with previous reports of chytrid abundance in coastal areas [56] and their ecological importance in Arctic systems [57].

Beyond Chytridiomycota, other non-Dikarya lineages also exhibited distinct habitat patterns. Microsporidia were widespread across most inland environments, except for hypersaline sediments. Aphelidea exhibited a stronger association with saline inland habitats, while Rozellida were detected in both marine and inland systems without a clear environmental preference. Although these groups are collectively referred to as non-Dikarya fungi, their ecological roles differ. To date, Microsporidia, Rozellida, and Aphelidea are primarily known as obligate parasites: Aphelidea typically infect algae [21]; Microsporidia target protists, invertebrates, and vertebrates [14]; and Rozellida are often described as hyperparasites of other fungi or fungi-like organisms [11, 20]. In contrast, Chytridiomycota include both parasitic and saprotrophic taxa, some of which are capable of degrading a wide range of substrates, thereby contributing to nutrient cycling in both aquatic and terrestrial environments [9, 11].

Interestingly, in our study we also identified the phylum-level clade NCLC1—previously described as parasites, specifically endoparasites, of marine diatoms [31]—as a recurring component of marine sediments. Its consistent presence in such habitats suggests that the ecological scope of this lineage may extend beyond its originally described host range, warranting further functional and taxonomic investigation of this group. Together, our findings reveal that non-Dikarya lineages are widespread, which may suggest—though does not demonstrate—that they could fulfill diverse and underappreciated ecological functions across aquatic and terrestrial environments.

### Habitat Preferences Among Related ASVs

When examining the distribution of the most abundant non-dikaryotic fungal groups at both clade and ASV levels (Fig. 6), we observe that while ASVs are usually restricted to specific related systems, individual clades rarely exhibit strict habitat specificity. Most clades are compositionally heterogeneous and contain ASVs associated with diverse environmental types. This pattern, previously reported in focused studies on Rozellida and Aphelidea [23], is now confirmed across a much broader dataset. The presence of phylogenetically related but ecologically distinct ASVs within a clade suggests that closely related lineages may have undergone niche divergence, possibly reflecting host shifts, substrate specialization (in the case of chytrid saprotrophs), or adaptive responses to different environmental conditions. A similar pattern has been previously discussed for Microsporidia [17], supporting the view that ecological breadth at higher taxonomic levels may emerge from the coexistence of multiple, highly specialized taxa.

### Non-Dikarya Fungi in Extreme Environments

Non-Dikarya lineages are frequently detected in extreme environments such as polar systems, hypersaline lakes and sediments, and deep-sea sediments. Their consistent presence across such habitats suggests that tolerance to extreme conditions is not limited to Dikarya (Ascomycota and Basidiomycota), despite most studies on extremophilic fungi being focused on these groups [58]. Non-Dikarya fungi may also possess ecological and physiological traits that allow them to persist and function under harsh conditions. One possible adaptation is the formation of resting or dormant spores, which have been described in aphelids [59], rozellids [19], and chytrids [9]. Resting spores likely enable survival under prolonged stress and may explain the persistence of non-dikaryotic fungi in sediments and other low-activity habitats with limited hosts. Environmental pressures such as low temperature or salinity fluctuations likely select for fungi with resistant life stages. Indeed, environmental conditions can strongly structure fungal distributions at the clade level. In this sense, we have unveiled the Aphelidea APH03 clade (previously referred to as GS16 by Tedersoo et al. (2017) [54]) and the novel Microsporidia clade MIC05 as clearly associated with hypersaline lake environments. This pattern suggests that high salinity may act as a strong environmental filter, limiting the distribution of entire clades. This, combined with high phylogenetic novelty, highlights how extreme environments can act as reservoirs of undescribed fungal diversity.

## Conclusion

By using near full-length 18S rRNA gene sequences as a phylogenetic backbone, we were able to achieve accurate placement of short V4 reads from environmental samples. According to our data, the V4 region is informative up to the order level in Chytridimycota and can be used to define clades in other non-Dikarya fungi. From an ecological perspective, our data reinforce that fungi are present across a wide range of environments, with higher relative abundance and group diversity in inland habitats compared to marine systems. Although Ascomycota and Basidiomycota are the most well known and extensively studied fungal phyla, they absolutely dominate fungal communities only in open oceanic waters, which also showed the lowest relative abundance of fungal sequences. Conversely, non-Dikarya groups—especially Chytridiomycota, Microsporidia, Rozellida, and Aphelidea—are widely distributed across diverse ecosystems and, in many cases, are even more abundant than Ascomycota and Basidiomycota. Given that these non-Dikarya fungi are predominantly known as parasites, we can infer an important ecological role of fungal parasitism in natural habitats. However, for some non-Dikarya groups, alternative lifestyles such as other forms of symbiosis or saprotrophy may also exist but remain undescribed. At finer taxonomic scales, non-dikaryotic fungal clades exhibit ecological diversity. Most lineages include phylogenetically related ASVs that are each restricted to specific habitats, suggesting that closely related taxa have undergone ecological specialization, potentially driven by host or substrate differences. This pattern, now confirmed across a large dataset, supports earlier findings from focused studies on Chytridiomycota and Microsporidia plus Rozellida. Non-dikaryotic fungal diversity is also prominent in extreme environments, including polar systems, hypersaline lakes, and deep-sea sediments. The persistence of these lineages in harsh conditions may be enabled by resistant life stages such as resting spores, and by ecological traits not yet fully understood. Our findings highlight extreme environments as important reservoirs of uncharacterized fungal diversity. Finally, our results highlight the ongoing challenges in the taxonomic resolution of fungi. A minor fraction of ASVs could only be classified as “InSedFungi” or Opisthokonta incertae sedis, indicating the presence of fungal diversity that remains to be described. This gap illustrates the need for broader taxon sampling, improved reference frameworks, and consistent naming conventions to integrate emerging environmental diversity into fungal systematics.

## Supplementary Information

Below is the link to the electronic supplementary material.

ESM 1 (DOCX 19.9 MB)

## Data Availability

The phyloseq object, along with all sequence alignments used for phylogenetic analyses, is available on GitHub (https://github.com/MassanaLab/fungi_global_metabarcoding).

## References

[1] Martínez AT, Speranza M, Ruiz-Dueñas FJ et al (2005) Biodegradation of lignocellulosics: microbial, chemical, and enzymatic aspects of the fungal attack of lignin. Int Microbiol Off J Span Soc Microbiol 8:195–204 16200498

[2] Grinhut T, Hadar Y, Chen Y (2007) Degradation and transformation of humic substances by saprotrophic fungi: processes and mechanisms. Fungal Biol Rev 21:179–189. 10.1016/j.fbr.2007.09.003

[3] Smith SE, Read D (2008) Mycorrhizal symbiosis, 3rd edn. Elsevier, Amsterdam. 10.1016/B978-0-12-370526-6.X5001-6

[4] Richards TA, Jones MDM, Leonard G, Bass D (2012) Marine fungi: their ecology and molecular diversity. Annu Rev Mar Sci 4:495–522. 10.1146/annurev-marine-120710-100802 10.1146/annurev-marine-120710-100802 22457985

[5] Zhang C, Meng Y, Zhao M et al (2024) Advances and mechanisms of fungal symbionts in improving the salt tolerance of crops. Plant Sci 349:112261. 10.1016/j.plantsci.2024.112261 39270825 10.1016/j.plantsci.2024.112261

[6] Grossart H-P, Wurzbacher C, James TY, Kagami M (2016) Discovery of dark matter fungi in aquatic ecosystems demands a reappraisal of the phylogeny and ecology of zoosporic fungi. Fungal Ecol 19:28–38. 10.1016/j.funeco.2015.06.004

[7] Reynolds NK, Jusino MA, Stajich JE, Smith ME (2022) Understudied, underrepresented, and unknown: methodological biases that limit detection of early diverging fungi from environmental samples. Mol Ecol Resour 22:1065–1085. 10.1111/1755-0998.13540 34695878 10.1111/1755-0998.13540

[8] James TY, Rokas A (2025) Use their names: there are no basal, lower, or early diverging fungi. Mycologia 117:246–254. 10.1080/00275514.2025.2460003 40020155 10.1080/00275514.2025.2460003 PMC11903137

[9] Powell MJ, Letcher PM (2014) 6 Chytridiomycota, Monoblepharidomycota, and Neocallimastigomycota. In: McLaughlin DJ, Spatafora JW (eds) Systematics and Evolution. Springer, Berlin Heidelberg, Berlin, Heidelberg, pp 141–175

[10] Ilicic D, Grossart H-P (2022) Basal parasitic fungi in marine food webs—a mystery yet to unravel. J Fungi 8:114. 10.3390/jof8020114 10.3390/jof8020114 PMC8874645 35205868

[11] Seto K, Simmons DR, Quandt CA, Frenken T, Dirks AC, Clemons RA, McKindles KM, McKay RM, James TY (2023) A combined microscopy and single-cell sequencing approach reveals the ecology, morphology, and phylogeny of uncultured lineages of zoosporic fungi. Mbio 14(4):e01313-23. 10.1128/mbio.01313-23 37486265 10.1128/mbio.01313-23 PMC10470594

[12] Kagami M, Miki T, Takimoto G (2014) Mycoloop: chytrids in aquatic food webs. Front Microbiol 22(5):166. 10.3389/fmicb.2014.00166 10.3389/fmicb.2014.00166 PMC4001071 24795703

[13] Grossart H-P, Van Den Wyngaert S, Kagami M et al (2019) Fungi in aquatic ecosystems. Nat Rev Microbiol 17:339–354. 10.1038/s41579-019-0175-8 30872817 10.1038/s41579-019-0175-8

[14] Bass D, Czech L, Williams BAP et al (2018) Clarifying the relationships between *Microsporidia* and *Cryptomycota*. J Eukaryot Microbiol 65:773–782. 10.1111/jeu.12519 29603494 10.1111/jeu.12519 PMC6282948

[15] Wijayawardene NN, Hyde KD, Mikhailov KV et al (2024) Classes and phyla of the kingdom fungi. Fungal Divers 128(1):1–165. 10.1007/s13225-024-00540-z

[16] Corsaro D, Wylezich C, Venditti D et al (2019) Filling gaps in the microsporidian tree: rDNA phylogeny of *Chytridiopsis typographi* (Microsporidia: Chytridiopsida). Parasitol Res 118:169–180. 10.1007/s00436-018-6130-1 30421347 10.1007/s00436-018-6130-1

[17] Gross M, Rajter Ľ, Mahé F et al (2024) O short-branch Microsporidia, where art thou? Identifying diversity hotspots for future sampling. Eur J Protistol 96:126119. 10.1016/j.ejop.2024.126119 39396432 10.1016/j.ejop.2024.126119

[18] Voigt K, James TY, Kirk PM et al (2021) Early-diverging fungal phyla: taxonomy, species concept, ecology, distribution, anthropogenic impact, and novel phylogenetic proposals. Fungal Divers 109:59–98. 10.1007/s13225-021-00480-y 34608378 10.1007/s13225-021-00480-y PMC8480134

[19] Letcher PM, Powell MJ (2018) A taxonomic summary and revision of Rozella (Cryptomycota). IMA Fungus 9:383–399. 10.5598/imafungus.2018.09.02.09 30622888 10.5598/imafungus.2018.09.02.09 PMC6317583

[20] Kagami M, Seto K, Nozaki D et al (2021) Single dominant diatom can host diverse parasitic fungi with different degree of host specificity. Limnol Oceanogr 66:667–677. 10.1002/lno.11631

[21] Karpov SA, Mamkaeva MA, Aleoshin VV, Nassonova E, Lilje O, Gleason FH (2014) Morphology, phylogeny, and ecology of the aphelids (Aphelidea, Opisthokonta) and proposal for the new superphylum Opisthosporidia. Front Microbiol 28(5):112. 10.3389/fmicb.2014.00112 10.3389/fmicb.2014.00112 PMC3975115 24734027

[22] Letcher PM, Powell MJ (2019) A taxonomic summary of Aphelidiaceae. IMA Fungus 10:4. 10.1186/s43008-019-0005-7 32647613 10.1186/s43008-019-0005-7 PMC7325670

[23] Quandt CA, Marino JA, Simmons DR et al (2023) Evaluating the diversity of the enigmatic fungal phylum Cryptomycota across habitats using 18S rRNA metabarcoding. Fungal Ecol 64:101248. 10.1016/j.funeco.2023.101248

[24] Comeau AM, Vincent WF, Bernier L, Lovejoy C (2016) Novel chytrid lineages dominate fungal sequences in diverse marine and freshwater habitats. Sci Rep 6:30120. 10.1038/srep30120 27444055 10.1038/srep30120 PMC4957111

[25] Rojas-Jimenez K, Wurzbacher C, Bourne EC et al (2017) Early diverging lineages within Cryptomycota and Chytridiomycota dominate the fungal communities in ice-covered lakes of the McMurdo Dry Valleys, Antarctica. Sci Rep 7:15348. 10.1038/s41598-017-15598-w 29127335 10.1038/s41598-017-15598-w PMC5681503

[26] Hurdeal VG, Gentekaki E, Hyde KD, Jeewon R (2021) Where are the basal fungi? Current status on diversity, ecology, evolution, and taxonomy. Biologia (Bratisl) 76:421–440. 10.2478/s11756-020-00642-4

[27] Zhang T, Ji Z, Chen X, Yu L (2023) Shotgun metagenomics reveals a diverse mycobiome in the seawater from a High Arctic fjord (Kongsfjorden, Svalbard). Environ Res 233:116437. 10.1016/j.envres.2023.116437 37331553 10.1016/j.envres.2023.116437

[28] Tedersoo L, Anslan S, Bahram M et al (2015) Shotgun metagenomes and multiple primer pair-barcode combinations of amplicons reveal biases in metabarcoding analyses of fungi. MycoKeys 10:1–43. 10.3897/mycokeys.10.4852

[29] Berney C, Mahé F, Henry N, et al (2023) EukBank 18S V4 dataset. 10.5281/zenodo.7804946

[30] Vaulot D, Sim CWH, Ong D et al (2022) MetaPR2: a database of eukaryotic 18S rRNA metabarcodes with an emphasis on protists. Mol Ecol Resour 22:3188–3201. 10.1111/1755-0998.13674 35762265 10.1111/1755-0998.13674 PMC9796713

[31] Chambouvet A, Monier A, Maguire F et al (2019) Intracellular infection of diverse diatoms by an evolutionary distinct relative of the fungi. Curr Biol 29:4093-4101.e4. 10.1016/j.cub.2019.09.074 31735677 10.1016/j.cub.2019.09.074

[32] Guillou L, Bachar D, Audic S et al (2012) The protist ribosomal reference database (PR2): a catalog of unicellular eukaryote small sub-unit rRNA sequences with curated taxonomy. Nucleic Acids Res 41(D1):D597–D604. 10.1093/nar/gks1160 23193267 10.1093/nar/gks1160 PMC3531120

[33] Altschul SF, Gish W, Miller W et al (1990) Basic local alignment search tool. J Mol Biol 215:403–410. 10.1016/S0022-2836(05)80360-2 2231712 10.1016/S0022-2836(05)80360-2

[34] Stoeck T, Bass D, Nebel M et al (2010) Multiple marker parallel tag environmental DNA sequencing reveals a highly complex eukaryotic community in marine anoxic water. Mol Ecol 19(s1):21–31. 10.1111/j.1365-294X.2009.04480.x 20331767 10.1111/j.1365-294X.2009.04480.x

[35] Balzano S, Abs E, Leterme S (2015) Protist diversity along a salinity gradient in a coastal lagoon. Aquat Microb Ecol 74:263–277. 10.3354/ame01740

[36] Pebesma E, Bivand R (2023) Spatial data science: with applications in R, 1st edn. Chapman and Hall/CRC, New York

[37] R Core Team (2023) R: a language and environment for statistical computing. R foundation for statistical computing, Vienna, Austria. https://www.R-project.org/

[38] Pante E, Simon-Bouhet B (2013) Marmap: a package for importing, plotting and analyzing bathymetric and topographic data in R. PLoS ONE 8:e73051. 10.1371/journal.pone.0073051 24019892 10.1371/journal.pone.0073051 PMC3760912

[39] Berger SA, Stamatakis A (2011) Aligning short reads to reference alignments and trees. Bioinformatics 27:2068–2075. 10.1093/bioinformatics/btr320 21636595 10.1093/bioinformatics/btr320

[40] Barbera P, Kozlov AM, Czech L et al (2019) EPA-ng: massively parallel evolutionary placement of genetic sequences. Syst Biol 68:365–369. 10.1093/sysbio/syy054 30165689 10.1093/sysbio/syy054 PMC6368480

[41] Czech L, Barbera P, Stamatakis A (2020) Genesis and Gappa: processing, analyzing and visualizing phylogenetic (placement) data. Bioinformatics 36:3263–3265. 10.1093/bioinformatics/btaa070 32016344 10.1093/bioinformatics/btaa070 PMC7214027

[42] Nguyen L-T, Schmidt HA, Von Haeseler A, Minh BQ (2015) Iq-tree: a fast and effective stochastic algorithm for estimating maximum-likelihood phylogenies. Mol Biol Evol 32:268–274. 10.1093/molbev/msu300 25371430 10.1093/molbev/msu300 PMC4271533

[43] Katoh K, Standley DM (2013) MAFFT multiple sequence alignment software version 7: improvements in performance and usability. Mol Biol Evol 30:772–780. 10.1093/molbev/mst010 23329690 10.1093/molbev/mst010 PMC3603318

[44] Wickham H, Averick M, Bryan J et al (2019) Welcome to the tidyverse. J Open Source Softw 4(43):1686. 10.21105/joss.01686

[45] McMurdie PJ, Holmes S (2013) Phyloseq: an R package for reproducible interactive analysis and graphics of microbiome census data. PLoS ONE 8:e61217. 10.1371/journal.pone.0061217 23630581 10.1371/journal.pone.0061217 PMC3632530

[46] McLaren M (2025) speedyseq: faster implementations of phyloseq functions. https://github.com/mikemc/speedyseq

[47] Oksanen J, Simpson GL, Blanchet FG, et al (2025) vegan: community ecology package.10.32614/CRAN.package.vegan

[48] Gu Z (2022) Complex heatmap visualization. iMeta 1:e43. 10.1002/imt2.43 38868715 10.1002/imt2.43 PMC10989952

[49] Tedersoo L, Bahram M, Zinger L et al (2022) Best practices in metabarcoding of fungi: from experimental design to results. Mol Ecol 31:2769–2795. 10.1111/mec.16460 35395127 10.1111/mec.16460

[50] Massana R, Gobet A, Audic S et al (2015) Marine protist diversity in E uropean coastal waters and sediments as revealed by high-throughput sequencing. Environ Microbiol 17:4035–4049. 10.1111/1462-2920.12955 26119494 10.1111/1462-2920.12955

[51] Matsen FA, Kodner RB, Armbrust EV (2010) pplacer: linear time maximum-likelihood and Bayesian phylogenetic placement of sequences onto a fixed reference tree. BMC Bioinformatics 11:538. 10.1186/1471-2105-11-538 21034504 10.1186/1471-2105-11-538 PMC3098090

[52] Richards TA, Leonard G, Wideman JG (2017) What defines the “kingdom” fungi? Microbiol Spectr 5(3):5.3.23. 10.1128/microbiolspec.FUNK-0044-2017 10.1128/microbiolspec.funk-0044-2017 PMC11687502 28643626

[53] Bartošová-Sojková P, Butenko A, Richtová J et al (2024) Inside the host: understanding the evolutionary trajectories of intracellular parasitism. Annu Rev Microbiol 78:39–59. 10.1146/annurev-micro-041222-025305 38684082 10.1146/annurev-micro-041222-025305

[54] Tedersoo L, Bahram M, Puusepp R et al (2017) Novel soil-inhabiting clades fill gaps in the fungal tree of life. Microbiome 5:42. 10.1186/s40168-017-0259-5 28388929 10.1186/s40168-017-0259-5 PMC5385062

[55] Tedersoo L, Hosseyni Moghaddam MS, Mikryukov V et al (2024) EUKARYOME: the rRNA gene reference database for identification of all eukaryotes. Database 2024:baae043. 10.1093/database/baae043 38865431 10.1093/database/baae043 PMC11168333

[56] Peng X, Amend AS, Baltar F et al (2024) Planktonic marine fungi: a review. J Geophys Res Biogeosciences 129:e2023JG007887. 10.1029/2023JG007887

[57] Hassett BT, Gradinger R (2016) Chytrids dominate arctic marine fungal communities. Environ Microbiol 18:2001–2009. 10.1111/1462-2920.13216 26754171 10.1111/1462-2920.13216

[58] Sahay S (2022) Extremophilic fungi: ecology, physiology and applications. Springer Nature Singapore, Singapore

[59] Letcher PM, Lopez S, Schmieder R et al (2013) Characterization of *Amoeboaphelidium protococcarum*, an algal parasite new to the Cryptomycota isolated from an outdoor algal pond used for the production of biofuel. PLoS ONE 8:e56232. 10.1371/journal.pone.0056232 23437098 10.1371/journal.pone.0056232 PMC3577820

