# Supplementary material for: Non-Dikarya Fungal Clades Are Everywhere: What 18S rRNA Gene Metabarcoding Reveals About Cross-System Distribution of Fungi
Source: Microb Ecol. 2025 Nov 24;89(1):4. doi: 10.1007/s00248-025-02642-w (PMC12743110; doi:10.1007/s00248-025-02642-w)
Supplement: Supplementary file 1 — (DOCX 19.9 MB) [file 248_2025_2642_MOESM1_ESM.pdf]

Supplementary Information (SI) for the paper submitted to

Microbial Ecology:

Basal fungal clades are everywhere: what 18S rRNA gene metabarcoding reveals about cross-system distribution of fungi

Sofiya Bondarenko[1], Aleix Obiol[1], Emilio O. Casamayor[2], Ramon Massana[1]

[1] Department of Marine Biology and Oceanography, Institut de Ciències del Mar (ICM-CSIC), Barcelona, Catalonia, Spain.

[2] Ecology of the Global Microbiome-Department of Ecology and Complexity, Centre of Advanced Studies of Blanes (CEAB-CSIC), Blanes, Spain

***Corresponding authors:**

Sofiya Bondarenko (sofiya@icm.csic.es) and Ramon Massana (ramonm@icm.csic.es)

Institut de Ciències del Mar (ICM-CSIC)

Passeig Marítim de la Barceloneta, 37-49

08003 Barcelona, Catalonia, Spain

Fig. S1. Reference phylogenetic tree of fungi and other Opisthokonta based on long 18S rRNA gene sequences. The tree was built using IQ-TREE with SYM+R10 model using 1031 Opisthokonta sequences and 12 outgroup sequences (clustered at 99% identity and 95% for Metazoa). Support values were estimated using 1000 SH-aLRT and 1000 UFBoot replicates.

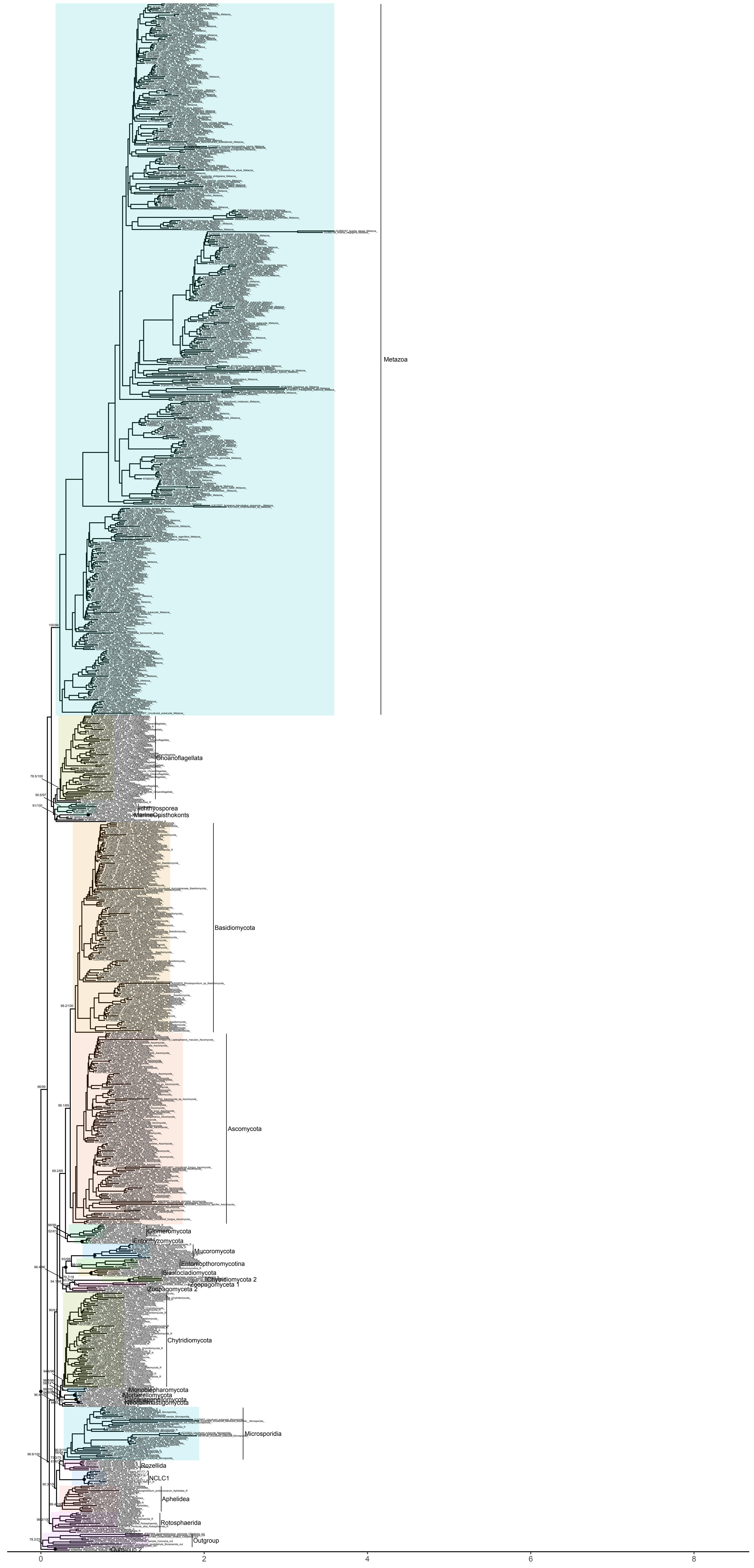

Metazoa
Choanoflagellata
Ichthyosporea
MarineOpisthokonts
Basidiomycota
Ascomycota
Glomeromycota
Entorrhizomycota
Mucoromycota
Entomophthoromycotina
Blastocladiomycota
Chytridiomycota 2
Zoopagomycota 1
Zoopagomycota 2
Chytridiomycota
Monoblepharomycota
Mortierellomycota
Calcarisporiellomycota
Neocallimastigomycota
Microsporidia
Rozellida
NCLC1
Aphelidea
Rotosphaerida
Outgroup
Outgroup 2
0
2
4
6
8

Fig. S2. World maps showing the geographic distribution of samples, grouped and displayed separately by habitat type.

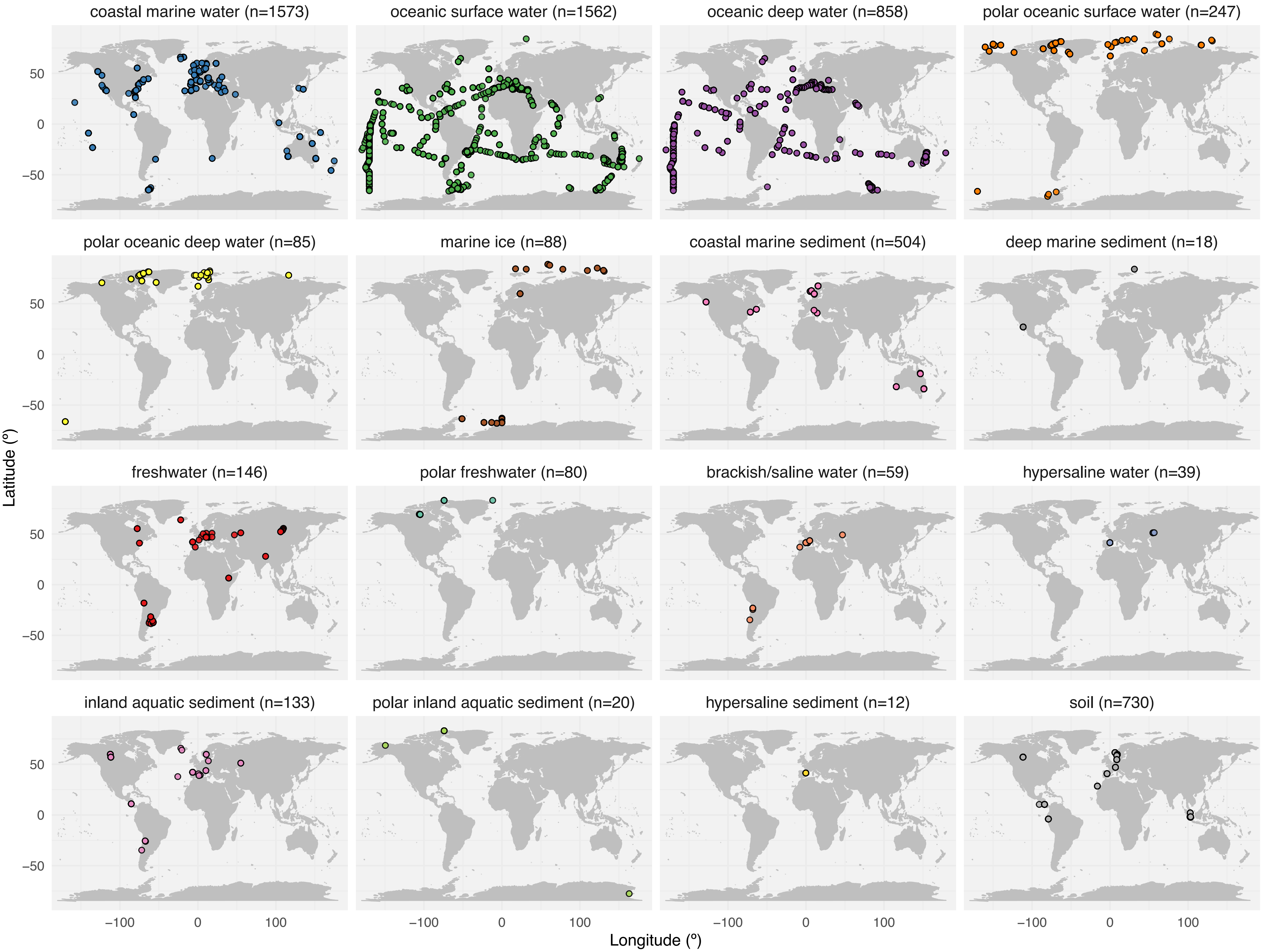
coastal marine water (n=1573)
oceanic surface water (n=1562)
oceanic deep water (n=858)
polar oceanic surface water (n=247)
polar oceanic deep water (n=85)
marine ice (n=88)
coastal marine sediment (n=504)
deep marine sediment (n=18)
freshwater (n=146)
polar freshwater (n=80)
brackish/saline water (n=59)
hypersaline water (n=39)
inland aquatic sediment (n=133)
polar inland aquatic sediment (n=20)
hypersaline sediment (n=12)
soil (n=730)
Latitude (°)
50
0
−50
Longitude (°)
−100
0
100

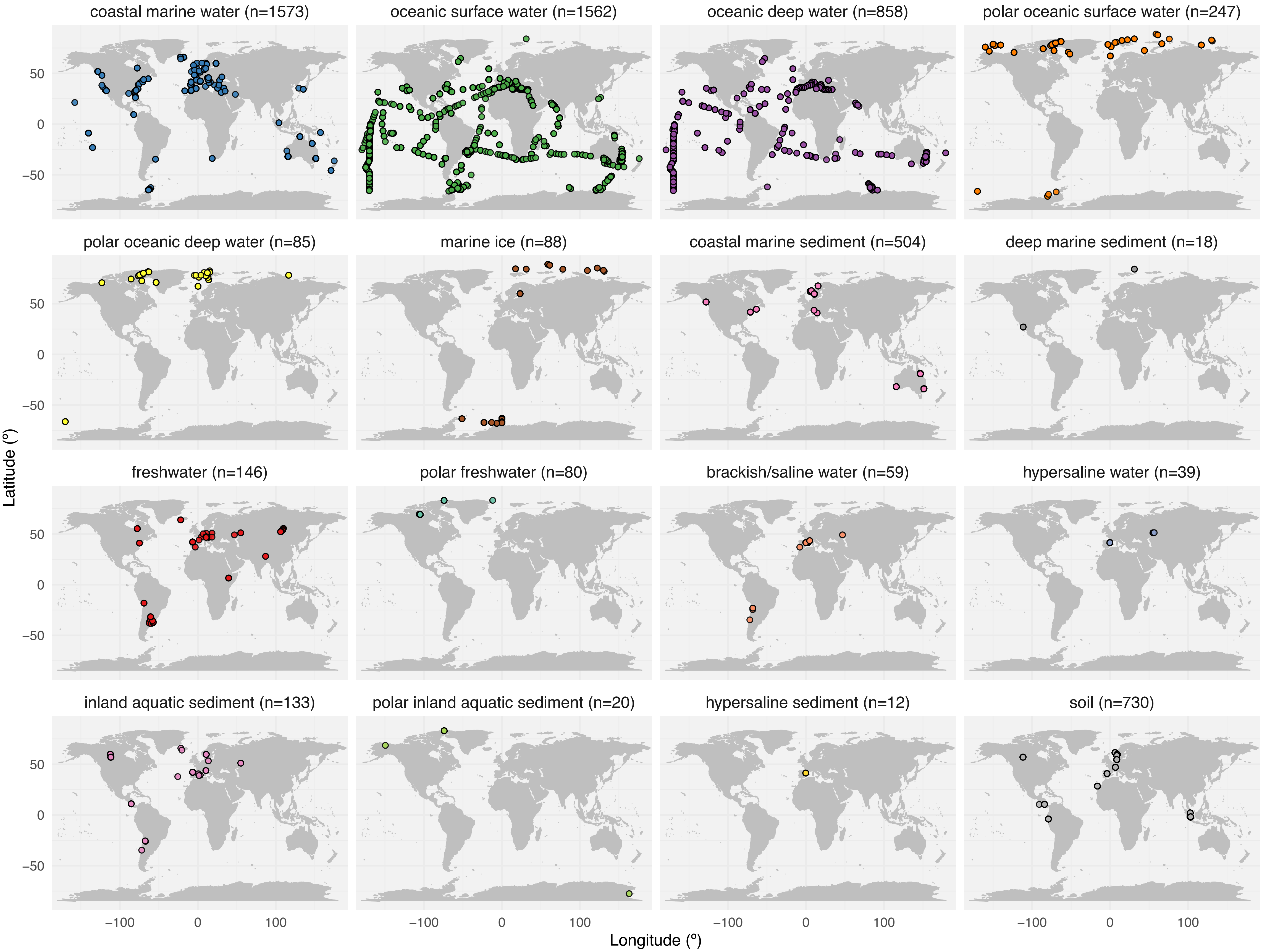

coastal marine water (n=1573)
oceanic surface water (n=1562)
oceanic deep water (n=858)
polar oceanic surface water (n=247)
polar oceanic deep water (n=85)
marine ice (n=88)
coastal marine sediment (n=504)
deep marine sediment (n=18)
freshwater (n=146)
polar freshwater (n=80)
brackish/saline water (n=59)
hypersaline water (n=39)
inland aquatic sediment (n=133)
polar inland aquatic sediment (n=20)
hypersaline sediment (n=12)
soil (n=730)
Latitude (°)
Longitude (°)
50
0
−50
−100
0
100

Fig. S3. NMDS of samples based on fungal ASVs using Bray–Curtis dissimilarities for three main habitat categories. For this analysis, we considered only those samples that contained a minimum of 100 fungal ASV reads.

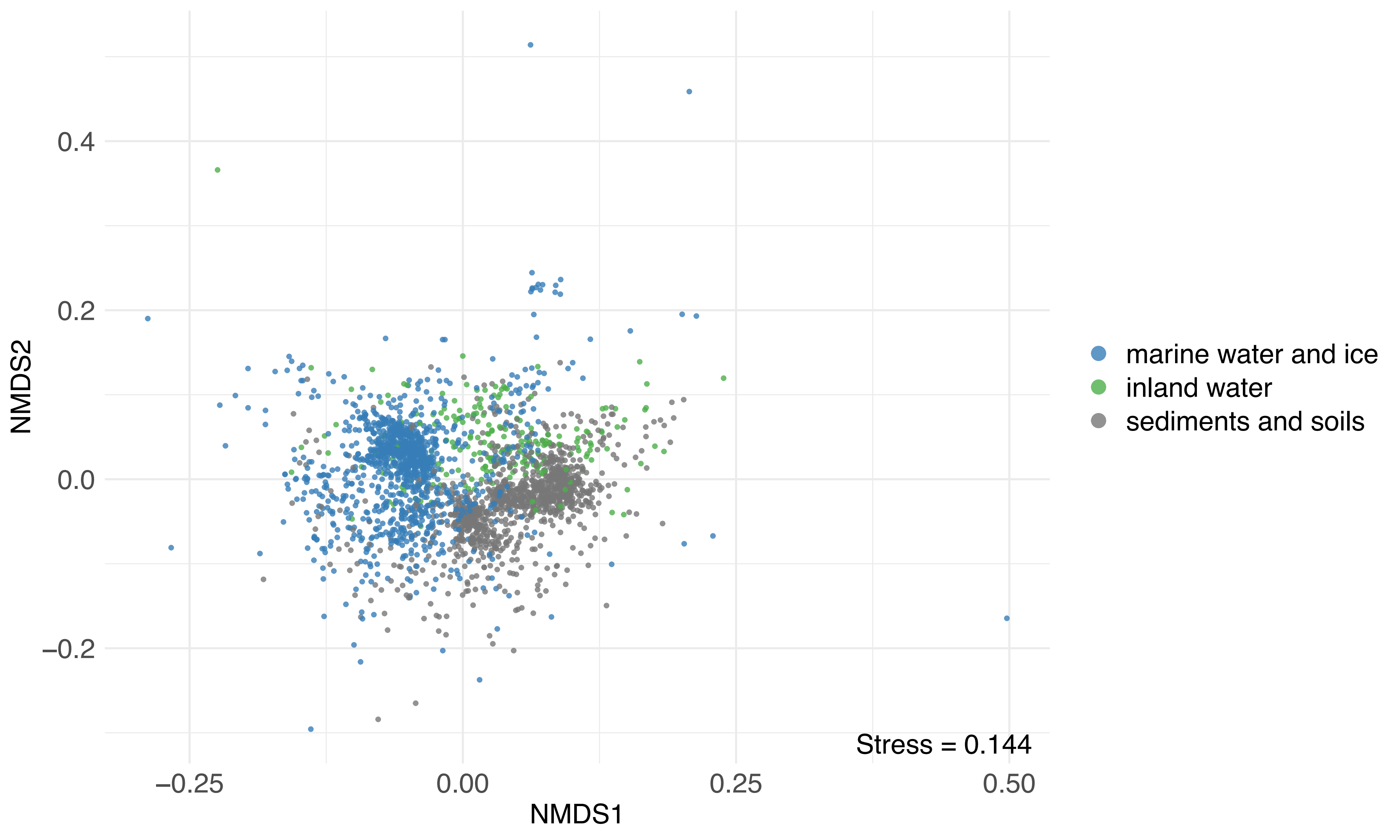

NMDS2
0.4
0.2
0.0
−0.2
NMDS1
−0.25
0.00
0.25
0.50
Stress = 0.144
marine water and ice
inland water
sediments and soils

Fig. S4. Relative read abundance of the main fungal groups in various habitats. Each dot represents the pooled relative read abundance of all ASVs assigned to a given group within a sample. Values are shown on a logarithmic scale, with the lowest positive value in the dataset used as a pseudocount. Boxes in plots represent the interquartile range (IQR), with the median line inside. Whiskers extend 1.5 times the IQR and individual points beyond this range represent outliers.

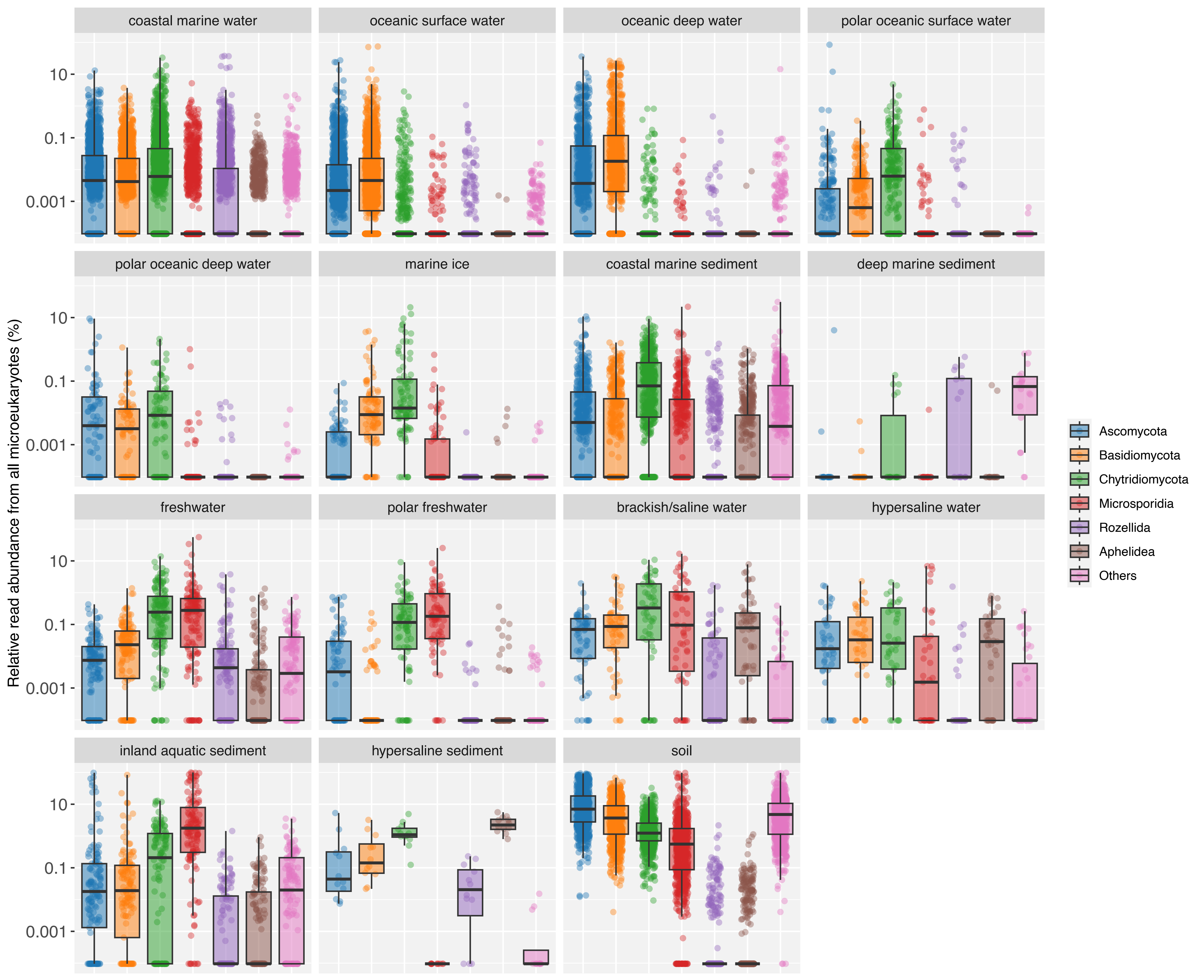

coastal marine water
oceanic surface water
oceanic deep water
polar oceanic surface water
polar oceanic deep water
marine ice
coastal marine sediment
deep marine sediment
freshwater
polar freshwater
brackish/saline water
hypersaline water
inland aquatic sediment
hypersaline sediment
soil
10
0.1
0.001
Relative read abundance from all microeukaryotes (%)
Ascomycota
Basidiomycota
Chytridiomycota
Microsporidia
Rozellida
Aphelidea
Others

Fig. S5. Reference phylogenetic tree of Chytridiomycota based on the long reference 18S rRNA gene sequences. The tree was built using IQ-TREE with TIM2+F+R10 model. Support values were estimated using 1000 SH-aLRT and 1000 UFBoot replicates.

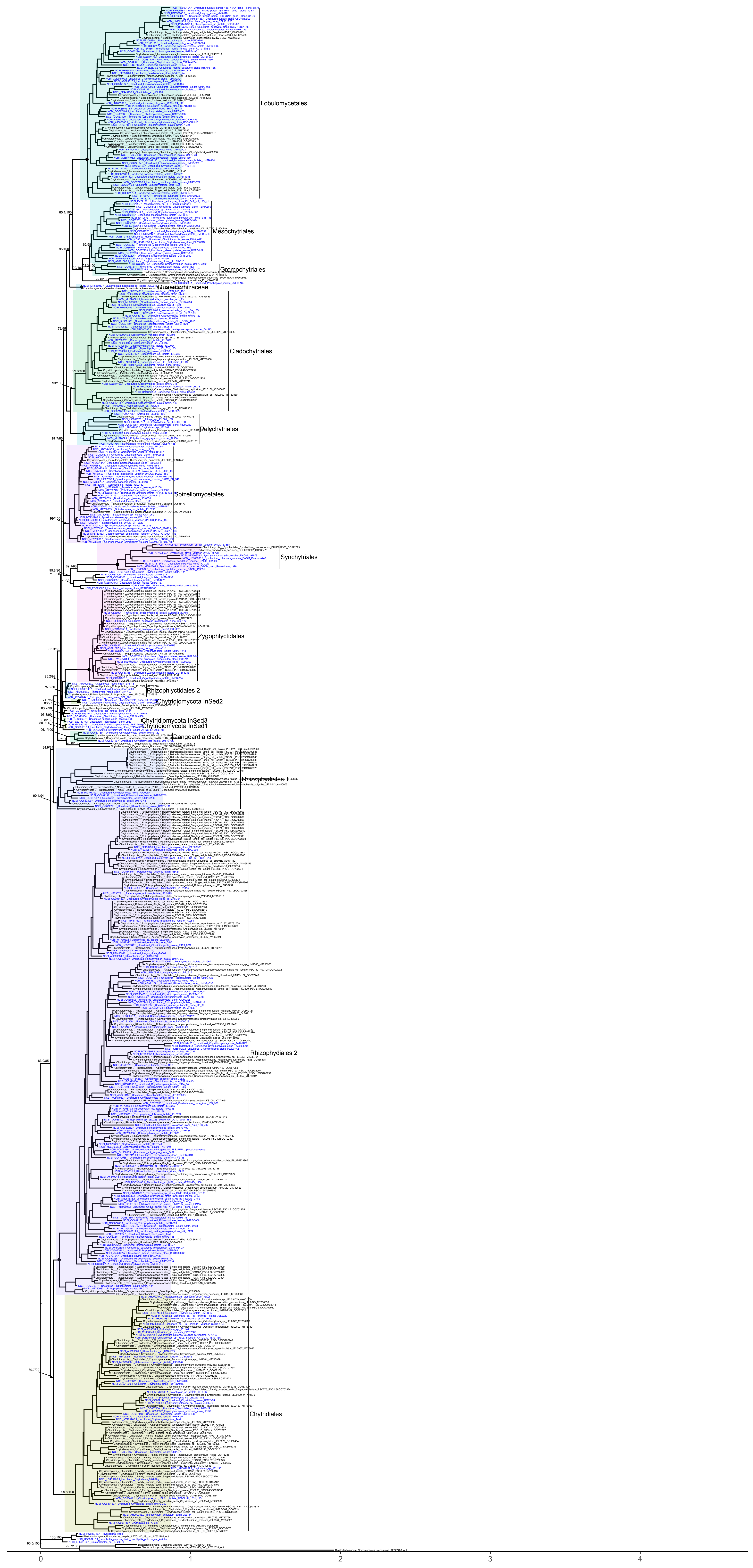
Lobulomycetales
Mesochytriales
Gromochytriales
Quaeritorhizaceae
Cladochytriales
Polychytriales
Spizellomycetales
Synchytriales
Zygophlyctidales
Rhizophlyctidales 2
Chytridiomycota InSed2
Chytridiomycota InSed3
Chytridiomycota InSed1
Dangeardia clade
Rhizophydiales 1
Rhizophydiales 2
Chytridiales
0
1
2
3
4

Fig. S6. Phylogenetic tree of Chytridiomycota based on the long and partial reference 18S rRNA gene sequences**.** In the tree, an alignment of complete reference sequences was first prepared and used to incorporate partial ASVs. ASVs present in more than 0.7% of samples and with a relative abundance greater than 0.1% were included. The tree was built using IQ-TREE with TIM2+F+R10 model. Support values were estimated using 1000 SH-aLRT and 1000 UFBoot replicates.

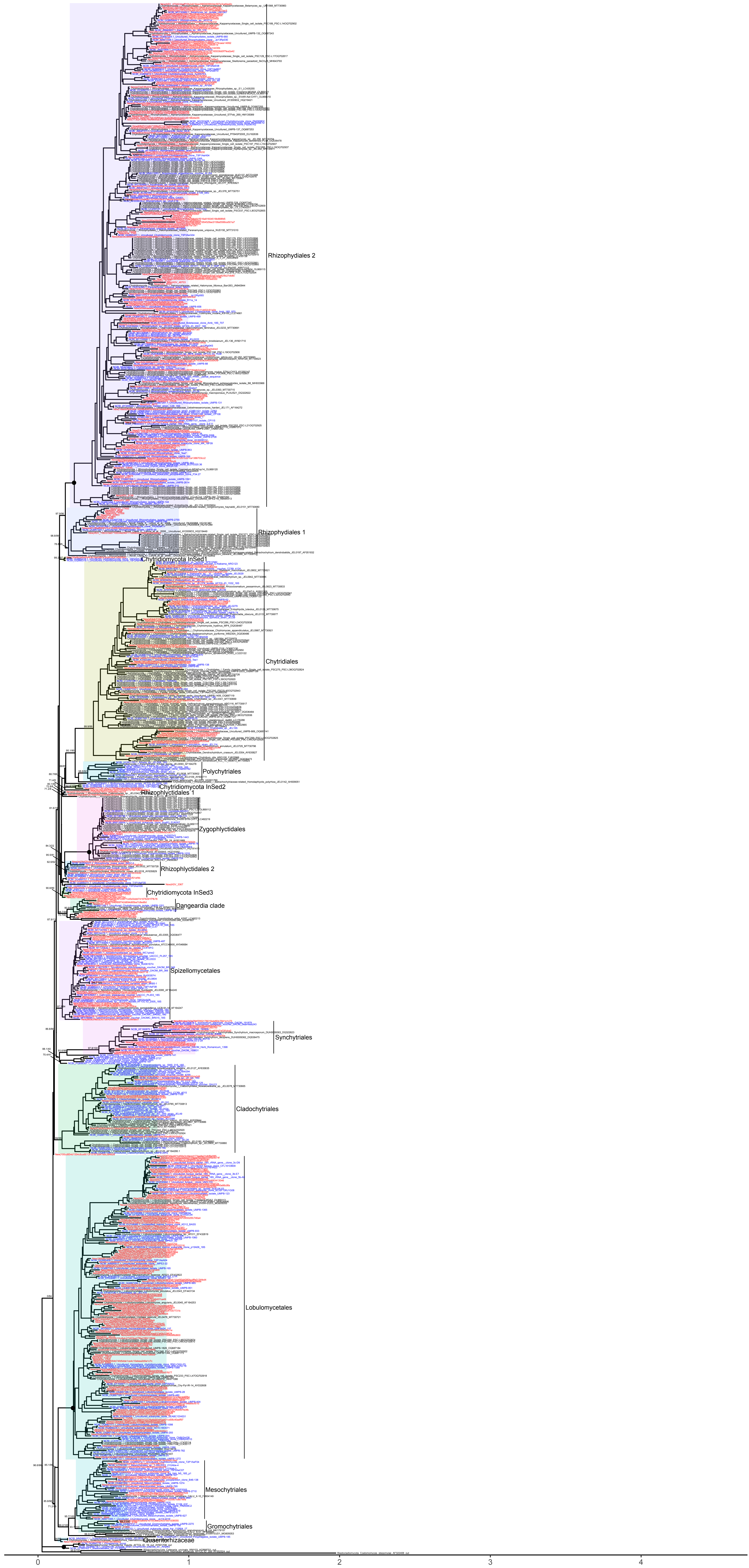

Rhizophydiales 2
Rhizophydiales 1
Chytridiomycota InSed1
Chytridiales
Polychytriales
Chytridiomycota InSed2
Rhizophlyctidales 1
Zygophlyctidales
Rhizophlyctidales 2
Chytridiomycota InSed3
Dangeardia clade
Spizellomycetales
Synchytriales
Cladochytriales
Lobulomycetales
Mesochytriales
Gromochytriales
Quaeritorhizaceae
0
1
2
3
4

Fig. S7. Reference phylogenetic tree of Microsporidia plus Rozellida based on the long reference 18S rRNA gene sequences. The tree was built using IQ-TREE with GTR+F+R7 model. Support values were estimated using 1000 SH-aLRT and 1000 UFBoot replicates.

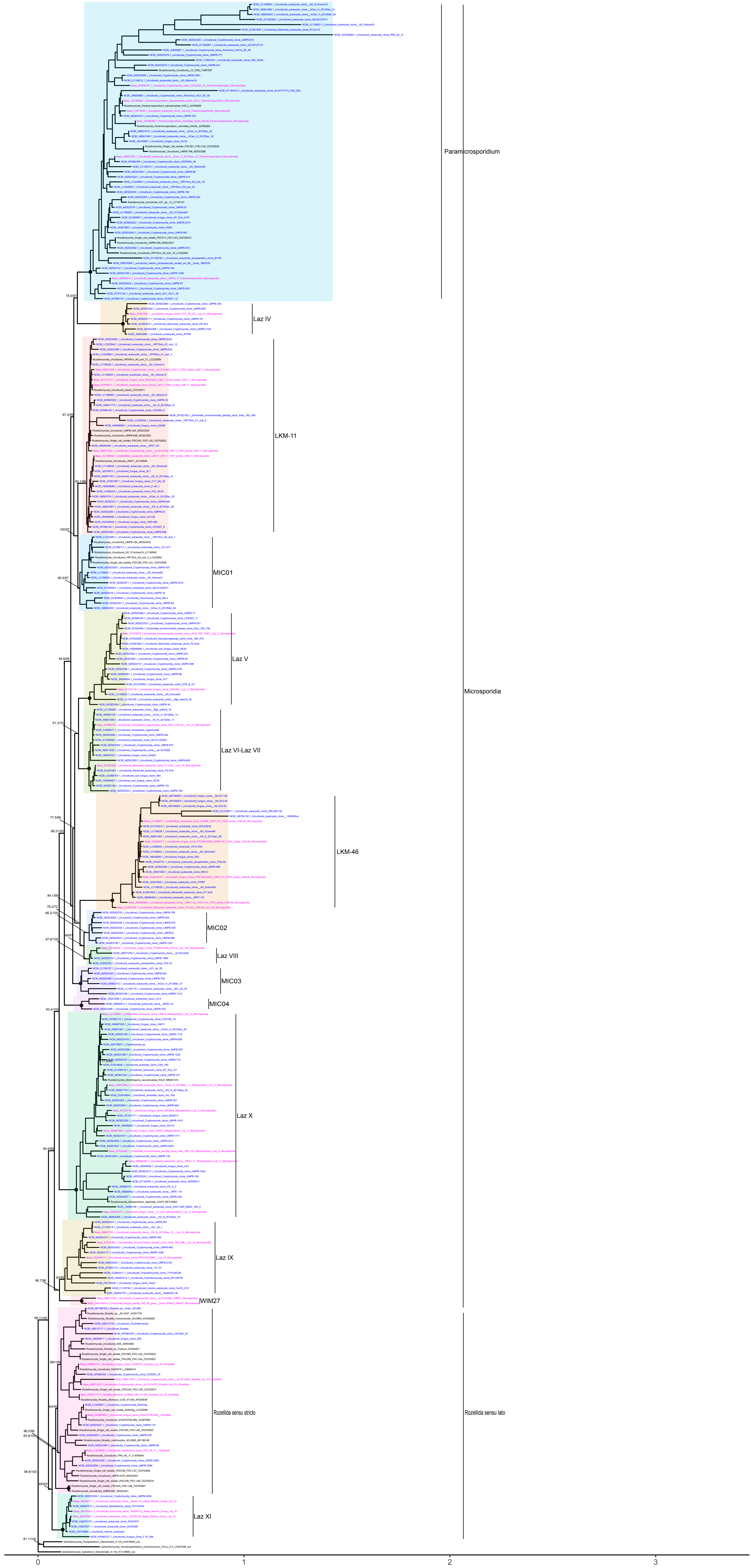
Paramicrosporidium
Laz IV
LKM-11
MIC01
Laz V
Laz VI-Laz VII
LKM-46
MIC02
Laz VIII
MIC03
MIC04
Laz X
Laz IX
WIM27
Microsporidia
Rozellida sensu stricto
Laz XI
Rozellida sensu lato
0
1
2
3

Fig S8. Phylogenetic tree of Microsporidia plus Rozellida based on the long and partial reference 18S rRNA gene sequences**.** In the tree, an alignment of complete reference sequences was first prepared and used to incorporate partial ASVs. ASVs present in more than 1% of samples and with a relative abundance greater than 0.1% were included. The tree was built using IQ-TREE with GTR+F+R7 model. Support values were estimated using 1000 SH-aLRT and 1000 UFBoot replicates.

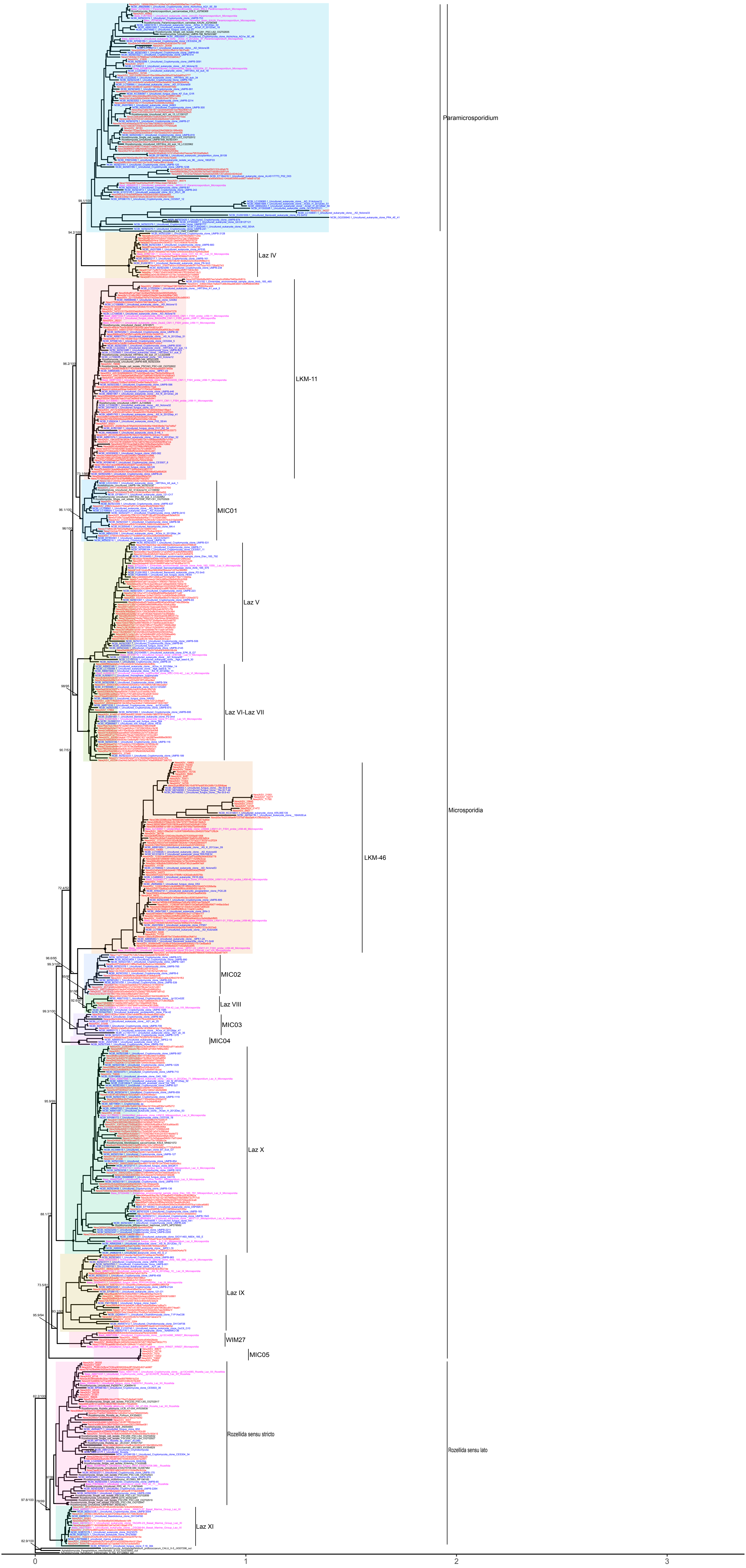
Paramicrosporidium
Laz IV
LKM-11
MIC01
Laz V
Laz VI-Laz VII
LKM-46
Microsporidia
MIC02
Laz VIII
MIC03
MIC04
Laz X
Laz IX
WIM27
MIC05
Rozellida sensu stricto
Rozellida sensu lato
Laz XI
0
1
2
3

Fig. S9. Reference phylogenetic tree of Aphelidea based on the long reference 18S rRNA gene sequences. The tree was built using IQ-TREE with TIM2+F+R4 model. Support values were estimated using 1000 SH-aLRT and 1000 UFBoot replicates.

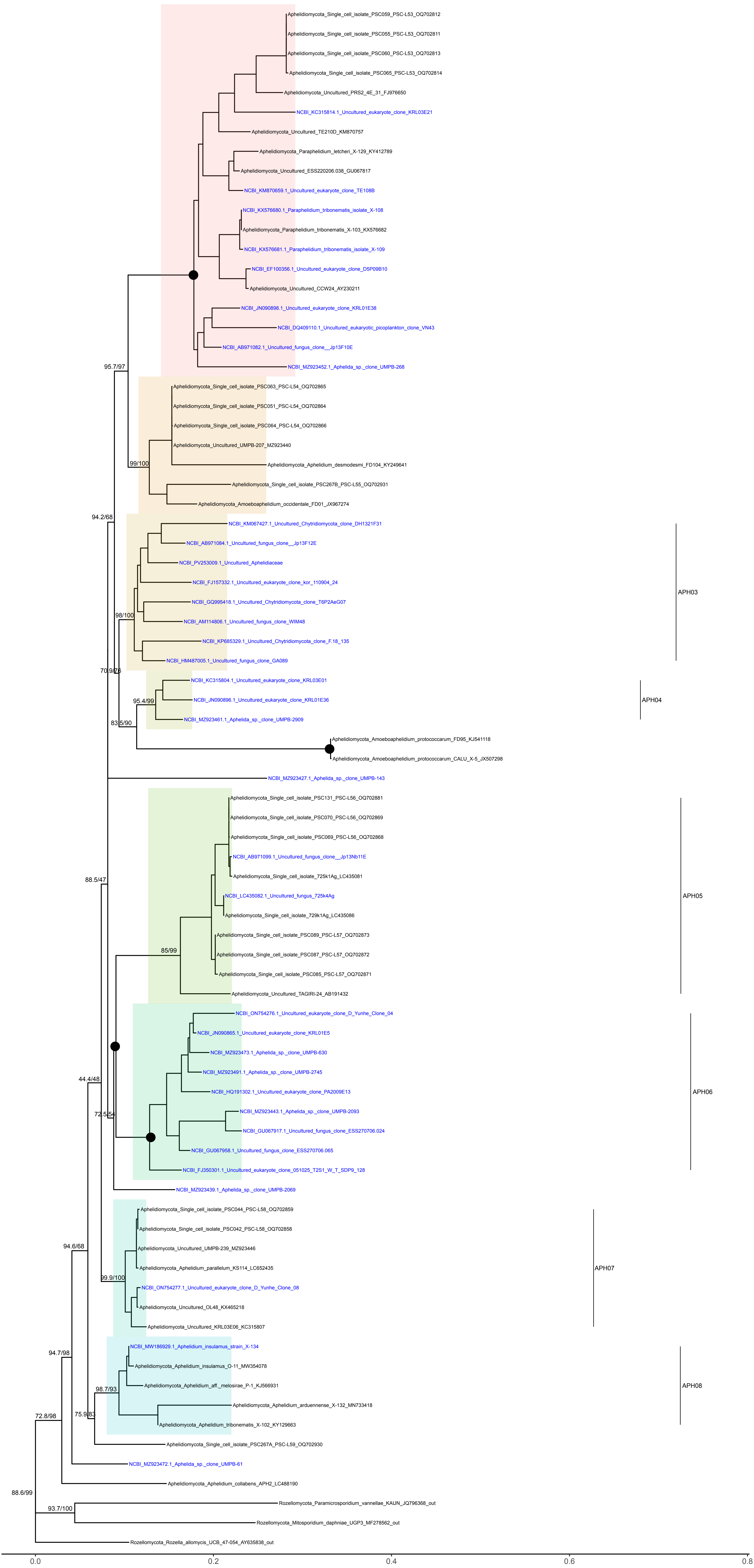

Aphelidiomycota_Single_cell_isolate_PSC059_PSC-L53_OQ702812
Aphelidiomycota_Single_cell_isolate_PSC055_PSC-L53_OQ702811
Aphelidiomycota_Single_cell_isolate_PSC060_PSC-L53_OQ702813
Aphelidiomycota_Single_cell_isolate_PSC065_PSC-L53_OQ702814
Aphelidiomycota_Uncultured_PRS2_4E_31_FJ976650
NCBI_KC315814.1_Uncultured_eukaryote_clone_KRL03E21
Aphelidiomycota_Uncultured_TE210D_KM870757
Aphelidiomycota_Paraphelidium_letcheri_X-129_KY412789
Aphelidiomycota_Uncultured_ESS220206.038_GU067817
NCBI_KM870659.1_Uncultured_eukaryote_clone_TE108B
NCBI_KX576680.1_Paraphelidium_tribonematis_isolate_X-108
Aphelidiomycota_Paraphelidium_tribonematis_X-103_KX576682
NCBI_KX576681.1_Paraphelidium_tribonematis_isolate_X-109
NCBI_EF100356.1_Uncultured_eukaryote_clone_D5P09B10
Aphelidiomycota_Uncultured_CCW24_AY230211
NCBI_JN090898.1_Uncultured_eukaryote_clone_KRL01E38
NCBI_DQ409110.1_Uncultured_eukaryotic_picoplankton_clone_VN43
NCBI_AB971082.1_Uncultured_fungus_clone__Jp13F10E
NCBI_MZ923452.1_Aphelida_sp._clone_UMPB-268
95.7/97
Aphelidiomycota_Single_cell_isolate_PSC063_PSC-L54_OQ702865
Aphelidiomycota_Single_cell_isolate_PSC051_PSC-L54_OQ702864
Aphelidiomycota_Single_cell_isolate_PSC064_PSC-L54_OQ702866
Aphelidiomycota_Uncultured_UMPB-207_MZ923440
99/100
Aphelidiomycota_Aphelidium_desmodesmi_FD104_KY249641
Aphelidiomycota_Single_cell_isolate_PSC267B_PSC-L55_OQ702931
Aphelidiomycota_Amoeboaphelidium_occidentale_FD01_JX967274
94.2/68
NCBI_KM067427.1_Uncultured_Chytridiomycota_clone_DH1321F31
NCBI_AB971084.1_Uncultured_fungus_clone__Jp13F12E
NCBI_PV253009.1_Uncultured_Aphelidiaceae
NCBI_FJ157332.1_Uncultured_eukaryote_clone_kor_110904_24
NCBI_GQ995418.1_Uncultured_Chytridiomycota_clone_T6P2AeG07
98/100
NCBI_AM114806.1_Uncultured_fungus_clone_WIM48
NCBI_KP685329.1_Uncultured_Chytridiomycota_clone_F.18_135
NCBI_HM487005.1_Uncultured_fungus_clone_GA089
APH03
70.9/76
NCBI_KC315804.1_Uncultured_eukaryote_clone_KRL03E01
95.4/99
NCBI_JN090896.1_Uncultured_eukaryote_clone_KRL01E36
NCBI_MZ923461.1_Aphelida_sp._clone_UMPB-2909
APH04
83.5/90
Aphelidiomycota_Amoeboaphelidium_protococcarum_FD95_KJ541118
Aphelidiomycota_Amoeboaphelidium_protococcarum_CALU_X-5_JX507298
NCBI_MZ923427.1_Aphelida_sp._clone_UMPB-143
Aphelidiomycota_Single_cell_isolate_PSC131_PSC-L56_OQ702881
Aphelidiomycota_Single_cell_isolate_PSC070_PSC-L56_OQ702869
Aphelidiomycota_Single_cell_isolate_PSC069_PSC-L56_OQ702868
NCBI_AB971099.1_Uncultured_fungus_clone__Jp13Nb11E
88.5/47
Aphelidiomycota_Single_cell_isolate_725k1Ag_LC435081
NCBI_LC435082.1_Uncultured_fungus_725k4Ag
Aphelidiomycota_Single_cell_isolate_729k1Ag_LC435086
APH05
Aphelidiomycota_Single_cell_isolate_PSC089_PSC-L57_OQ702873
85/99
Aphelidiomycota_Single_cell_isolate_PSC087_PSC-L57_OQ702872
Aphelidiomycota_Single_cell_isolate_PSC085_PSC-L57_OQ702871
Aphelidiomycota_Uncultured_TAGIRI-24_AB191432
NCBI_ON754276.1_Uncultured_eukaryote_clone_D_Yunhe_Clone_04
NCBI_JN090865.1_Uncultured_eukaryote_clone_KRL01E5
NCBI_MZ923473.1_Aphelida_sp._clone_UMPB-630
NCBI_MZ923491.1_Aphelida_sp._clone_UMPB-2745
44.4/48
NCBI_HQ191302.1_Uncultured_eukaryote_clone_PA2009E13
APH06
72.5/54
NCBI_MZ923443.1_Aphelida_sp._clone_UMPB-2093
NCBI_GU067917.1_Uncultured_fungus_clone_ESS270706.024
NCBI_GU067958.1_Uncultured_fungus_clone_ESS270706.065
NCBI_FJ350301.1_Uncultured_eukaryote_clone_051025_T2S1_W_T_SDP9_128
NCBI_MZ923439.1_Aphelida_sp._clone_UMPB-2069
Aphelidiomycota_Single_cell_isolate_PSC044_PSC-L58_OQ702859
Aphelidiomycota_Single_cell_isolate_PSC042_PSC-L58_OQ702858
94.6/68
Aphelidiomycota_Uncultured_UMPB-239_MZ923446
Aphelidiomycota_Aphelidium_parallelum_KS114_LC652435
APH07
99.9/100
NCBI_ON754277.1_Uncultured_eukaryote_clone_D_Yunhe_Clone_08
Aphelidiomycota_Uncultured_OL48_KX465218
Aphelidiomycota_Uncultured_KRL03E06_KC315807
NCBI_MW186929.1_Aphelidium_insulamus_strain_X-134
94.7/98
Aphelidiomycota_Aphelidium_insulamus_O-11_MW354078
Aphelidiomycota_Aphelidium_aff._melosirae_P-1_KJ566931
98.7/93
APH08
Aphelidiomycota_Aphelidium_arduennense_X-132_MN733418
75.9/83
72.8/98
Aphelidiomycota_Aphelidium_tribonematis_X-102_KY129663
Aphelidiomycota_Single_cell_isolate_PSC267A_PSC-L59_OQ702930
NCBI_MZ923472.1_Aphelida_sp._clone_UMPB-61
Aphelidiomycota_Aphelidium_collabens_APH2_LC488190
88.6/99
Rozellomycota_Paramicrosporidium_vannellae_KAUN_JQ796368_out
93.7/100
Rozellomycota_Mitosporidium_daphniae_UGP3_MF278562_out
Rozellomycota_Rozella_allomycis_UCB_47-054_AY635838_out
0.0
0.2
0.4
0.6
0.8

Fig. S10. Phylogenetic tree of Aphelidea based on the long and partial reference 18S rRNA gene sequences**.** In the tree, an alignment of complete reference sequences was first prepared and used to incorporate partial ASVs. All ASVs were included. The tree was built using IQ-TREE with SYM+R6 model. Support values were estimated using 1000 SH-aLRT and 1000 UFBoot replicates.

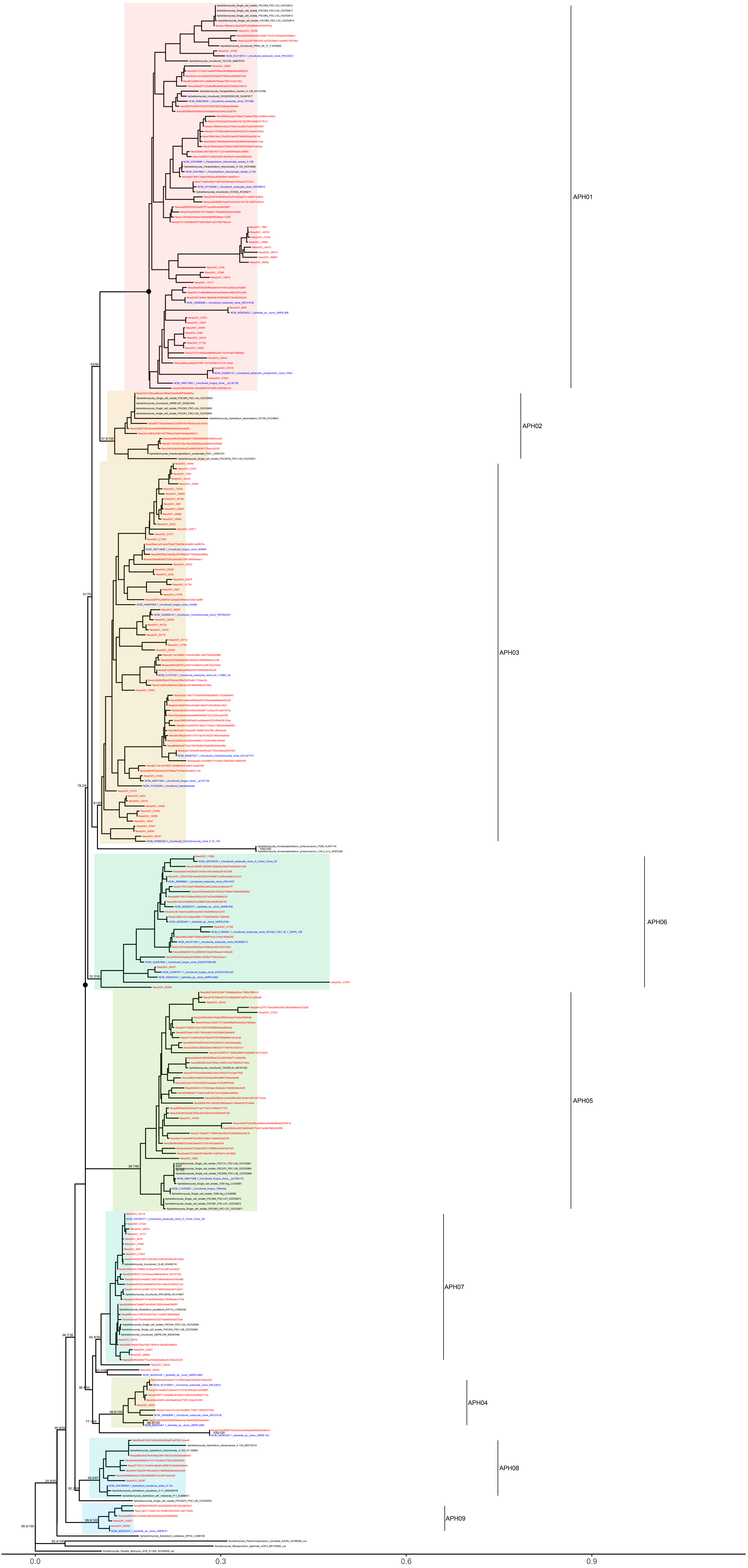
APH01
APH02
APH03
APH06
APH05
APH07
APH04
APH08
APH09
0.0
0.3
0.6
0.9
